# Defining the epitope of a blood–brain barrier crossing single domain antibody specific for the type 1 insulin-like growth factor receptor

**DOI:** 10.1038/s41598-021-83198-w

**Published:** 2021-02-19

**Authors:** Joey Sheff, Ping Wang, Ping Xu, Melanie Arbour, Luke Masson, Henk van Faassen, Greg Hussack, Kristin Kemmerich, Eric Brunette, Danica Stanimirovic, Jennifer J. Hill, John Kelly, Feng Ni

**Affiliations:** 1 Human Health Therapeutics Research Centre, National Research Council Canada, 100 Sussex Drive, Ottawa, ON K1A 0R6 Canada; 2 Human Health Therapeutics Research Centre, National Research Council Canada, 6100 Royalmount Avenue, Montreal, QC H4P 2R2 Canada; 3 Human Health Therapeutics Research Centre, National Research Council Canada, 1200 Montreal Road, Ottawa, ON K1A 0R6 Canada

**Keywords:** Biochemistry, Drug discovery, Structural biology

## Abstract

Ligand-activated signaling through the type 1 insulin-like growth factor receptor (IGF1R) is implicated in many physiological processes ranging from normal human growth to cancer proliferation and metastasis. IGF1R has also emerged as a target for receptor-mediated transcytosis, a transport phenomenon that can be exploited to shuttle biotherapeutics across the blood–brain barrier (BBB). We employed differential hydrogen–deuterium exchange mass spectrometry (HDX-MS) and nuclear magnetic resonance (NMR) to characterize the interactions of the IGF1R ectodomain with a recently discovered BBB-crossing single-domain antibody (sdAb), VHH-IR5, in comparison with IGF-1 binding. HDX-MS confirmed that IGF-1 induced global conformational shifts in the L1/FnIII-1/-2 domains and α-CT helix of IGF1R. In contrast, the VHH-IR5 sdAb-mediated changes in conformational dynamics were limited to the α-CT helix and its immediate vicinity (L1 domain). High-resolution NMR spectroscopy titration data and linear peptide scanning demonstrated that VHH-IR5 has high-affinity binding interactions with a peptide sequence around the C-terminal region of the α-CT helix. Taken together, these results define a core linear epitope for VHH-IR5 within the α-CT helix, overlapping the IGF-1 binding site, and suggest a potential role for the α-CT helix in sdAb-mediated transcytosis.

## Introduction

Human insulin like-growth factor receptor (IGF1R) is a member of a small family of transmembrane receptor tyrosine kinases (RTKs) that also includes the insulin receptor (IR) and a third member, the orphan insulin receptor-related receptor (IRR)^1^. Aside from IR, IGF1R is a secondary cell-signaling RTK involved in the regulation of glucose uptake and energy metabolism. IGF1R itself has important functions in fetal and prenatal growth and in cancer proliferation, by promoting the immortalization of transformed cells through binding to two insulin-like growth factors, IGF-1 and IGF-2^1–3^. Implication of IGF1R in cancer has prompted the development of anti-cancer strategies using neutralizing antibodies and small-molecule inhibitors of the RTK^4^. Both IR and, more recently, IGF1R have emerged as therapeutic targets for brain-specific neurological disorders, especially for drug-delivery across the blood–brain barrier (BBB)^5^.

Many diseases of the central nervous system (CNS) have limited treatment options because of the BBB, whose relative impermeability severely impedes the physical delivery of blood-borne drugs to the CNS. The BBB represents a particular hurdle to CNS delivery of antibody-based therapeutics^6^ and efficient crossing of large macromolecules must engage endocytotic pathways through a process called receptor-mediated transcytosis (RMT)^7,8^. To date, transferrin receptor (TfR)^9–11^, low-density lipoprotein (LDL)^12,13^, and insulin receptor (IR)^14,15^ have been explored as RMT targets, with IR-targeted therapies in advanced clinical trials^14^. However, targeting these common receptors has substantial development risks related to species-dependent expression in brain endothelial cells^16^, organ selectivity required for improved safety profiles^17^, as well as potential side effects associated with interfering with their normal physiological functions^18^. Our recent work has focused on IGF1R, based on its elevated expression in brain endothelial cells relative to peripheral tissue^19,20^, and on its established role in the delivery of one of its endogenous ligands, IGF-1, across the BBB^5,21^.

IGF1R is a heavily glycosylated transmembrane protein with a complex dimeric (αβ)_2_ architecture and with the two-chain (α and β) ectodomains held together by tertiary structural interactions along with inter-chain and intra-chain disulfide bonds^1,22^. The modular architecture of IGF1R includes a leucine-rich region (L1), a cysteine-rich region (CR), the second leucine-rich region (L2) and the N-terminal part of a type-III fibronectin-like domain FnIII-2 in the α-chain and the C-terminal part of FnIII-2 leading to two other fibronectin-like domains FnIII-1/3 in the β-chain of the ectodomain^1,22^. The intact FnIII-2 domain in the IGF1R precursor also contains a long insertion domain (ID) connecting the α- and β- chains generated by proteolytic cleavage at a site within ID^1^. Extensive structural studies of the IGF1R protein family have revealed a novel mechanism of receptor auto-inhibition and ligand-triggered receptor activation^22–24^. In such a mechanism, IGF-1 appears to engage only one L1-CR-L2 segment of the dimeric IGF1R along with one of the all-important α-CT helices exposed at the extreme C-termini of the α-chains (hence α-CTs) and to release an auto-inhibiting structure of the ectodomain leading to spontaneous receptor auto-phosphorylation^22–24^. A significant structural feature of the IGF1R/IGF-1 interaction is the malleability of the receptor architecture and the disparate roles played by the two α-CT helices in IGF1R, one as part of the binding site for IGF-1 and the second remaining apparently free with partial contacts with the L1′ and the FnIII-2 regions of the dimeric receptor^23,24^. The differing environment of the two α-CT helices elicited by IGF-1 binding to IGF1R has been identified as the structural origin^23,24^ of negative cooperativity observed for the IGF-1/IGF1R ligand-receptor system^25,26^.

We recently discovered a unique class of single-domain antibodies (sdAbs) specific for the IGF1R ectodomain^5,27^, among which the single-domain antibody IGF1R-5 (referred here to as VHH-IR5), does not appear to interfere with normal kinase functions of IGF1R and its interactions with the endogenous ligand^21^. As such, VHH-IR5 not only has the potential to act as a BBB-crossing shuttle, but may help decipher the fundamental mechanisms of cell signaling through IGF1R and receptor transcytosis. In this work, we focus on elucidating the mode of action of VHH-IR5, with a specific goal of defining its binding epitope on IGF1R in relation to the natural IGF1R ligand IGF-1. Results from differential hydrogen–deuterium exchange mass spectrometry (HDX-MS) and nuclear magnetic resonance (NMR) spectroscopy, and binding studies with IGF1R fragments outline an apparently shared binding site between the sdAb VHH-IR5 and the natural ligand IGF-1 convergent on the α-CT helix motif, an epitope on IGF1R not targeted, to the best of our knowledge, by other antibodies reported to date^28–31^.

## Results

### Section I: comparison of IGF-1 and VHH-IR5 binding and functional activation of IGF1R

**Figure 1:**
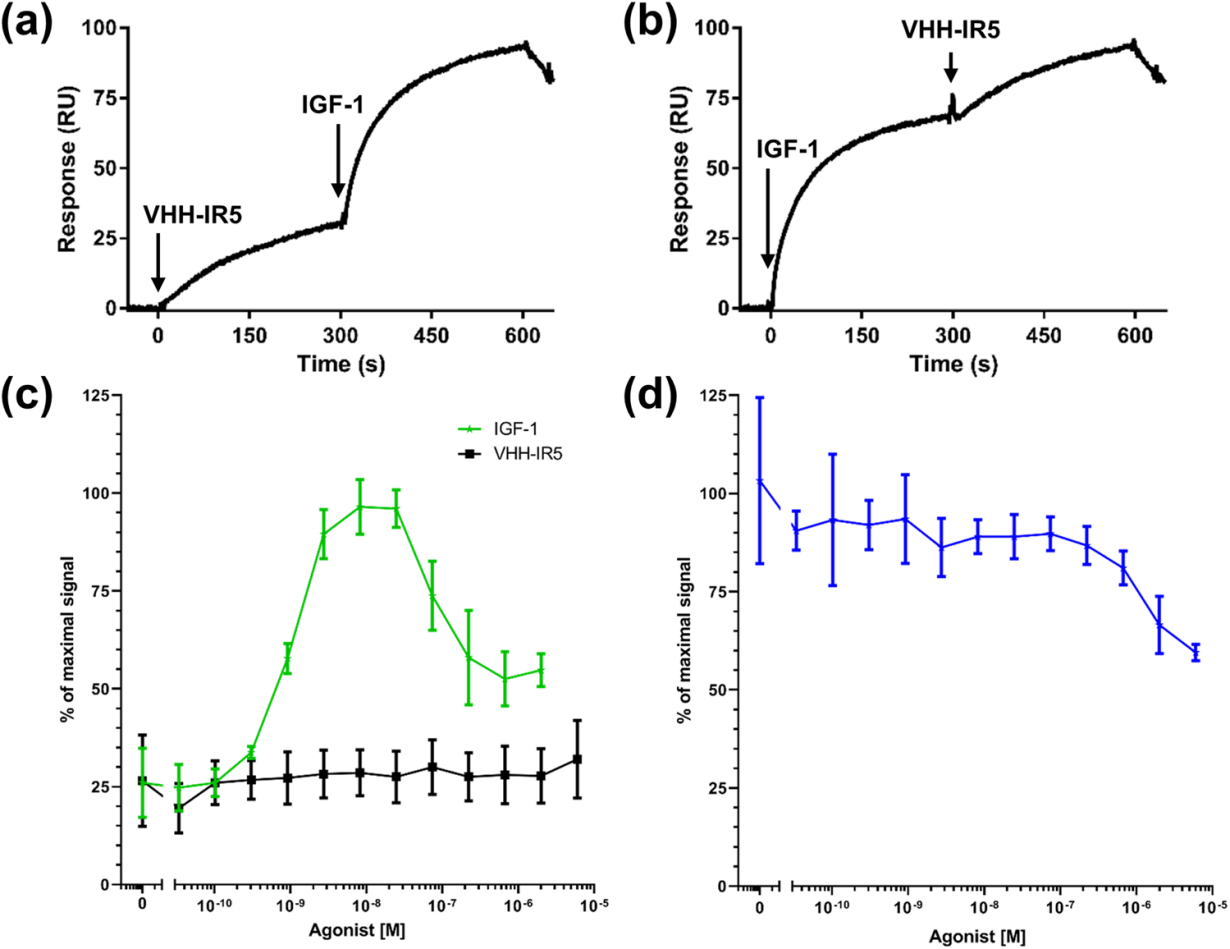
sdAb-IGF1R binding and effects on IGF1R signalling. (**a**) SPR sensorgrams showing the pre-loading of the sdAb VHH-IR5 to surface-immobilized human eIGF1R followed by the binding of human IGF-1. Experiments were carried out in duplicate with SEC-purified eIGF1R, IGF-1 and VHH-IR5 (see Fig. S1 of the Supplementary Materials). (**b**) SPR sensorgrams showing the pre-loading of human IGF-1 to surfaced immobilized eIGF1R followed by the binding of VHH-IR5. (**c**) Cell-based assays showing IGF1R activation by IGF-1 (green) and and lack of activation by VHH-IR5 (black) alone. (**d**) Cell-based assays showing effects of VHH-IR5 on IGF1R activation in the presence of 3 nM IGF-1 (blue).

Previous work has suggested that VHH-IR5, an IGF1R-binding sdAb (of the llama origin^27^) with BBB crossing activity, does not interfere with the interaction between IGF1R and its native ligand, IGF-1^21,27^. Figure 1a shows that saturation of surface-immobilized IGF1R ectodomain (eIGF1R) with VHH-IR5 does not preclude the subsequent binding of IGF-1. Reversing the order of pre-loading produced the same result (Fig. 1b). Qualitatively, pre-loading of either VHH-IR5 (Fig. 1a) or IGF-1 (Fig. 1b) also does not alter the shape of SPR sensograms of the other ligand (Fig. S2a,b). Thus, occupancy of eIGF1R by VHH-IR5 (or by IGF-1) has little effect on IGF-1 (or VHH-IR5) binding, outlining apparently independent and non-competitive binding sites on eIGF1R for these two ligands. Quantitatively, VHH-IR5 has a binding affinity of *K*_*D*_ = 0.6 nM for eIGF1R along with a slow rate of dissociation, *k*_*off*_ = 3.4 × 10^–4^ s^−1^, as seen by the characteristic plateau for the dissociation phase of the VHH-IR5 sdAb-eIGF1R complex (Fig. S2a). We measured an affinity of *K*_*D*_ = 8.2 nM, and a faster dissociation rate constant (8.2 × 10^–3^ s^−1^) for IGF-1 binding to the eIGF1R ectodomain (Fig. S2b), in agreement with previous reported values (*K*_*D*_ ~ 10–20 nM)^29^. VHH-IR5, therefore, has a higher affinity for eIGF1R as compared to IGF-1. In addition, VHH-IR5 is also specific for IGF1R as it does not bind the insulin receptor (Fig. S2c) in contrast to IGF-1 which interact with both (Fig. S2d).

Next, we demonstrated in a cell-based assay that VHH-IR5 binding does not induce significant activation of IGF1R, in sharp contrast to the effects of IGF-1 (Fig. 1c). Very importantly, increasing concentrations of VHH-IR5 had little further effects on the activating level of IGF-1, even when present up to > 500-fold molar excess (Fig. 1d), demonstrating essentially mutual independence of these two ligands. This finding is in agreement with previous studies that showed VHH-IR5 did not induce phosphorylation of the downstream kinase AKT^21^, which is the expected outcome of IGF1R activation by the endogenous ligand IGF-1^22,32^. Taken together, both biophysical binding (SPR) and cell-based assays indicate that VHH-IR5 and IGF-1 likely have independent binding sites on IGF1R and that VHH-IR5 binding is unlikely to alter the ligand-sensing capabilities of IGF1R on the cell-surface.

### Section II: comparison of IGF-1 and VHH-IR5 binding to IGF1R using HDX-MS

**Figure 2:**
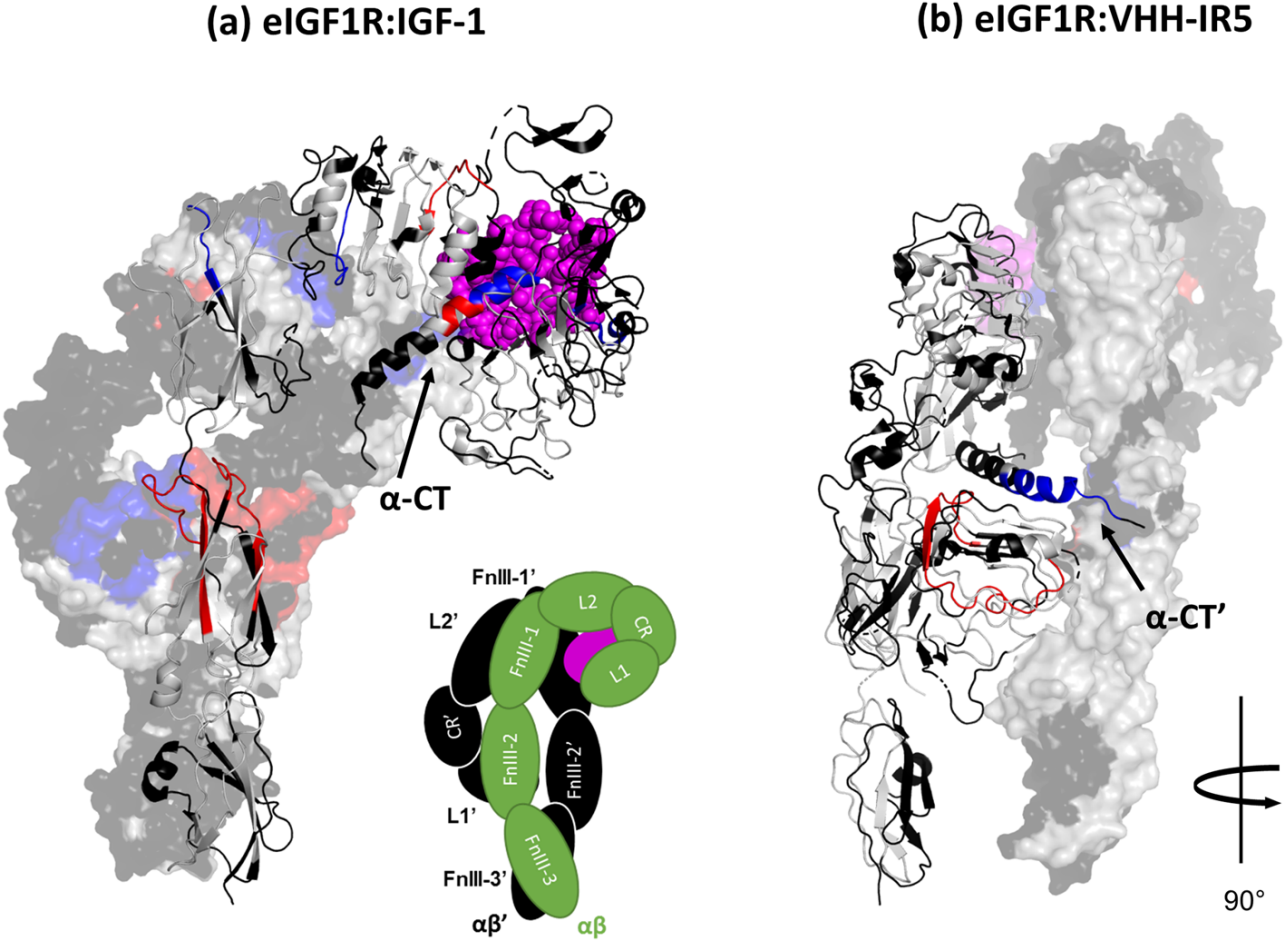
A global survey of HDX-MS profiles of eIGF1R in response to IGF-1 and VHH-IR5 binding. (**a**) IGF-1 response profile projected on a structural model of the 1:1 eIGF1R:IGF-1 complex (PDB: 6PYH). The (αβ) ′ monomer, except for α-CT′, is rendered as a transparent surface, while the (αβ) monomer and α-CT′ are shown as a ribbon cartoon. The schematic in the inset shows the domain organization of IGF1R where the (αβ) monomer is colored in green, and (αβ)′ in black. (**b**) VHH-IR5 response profile projected on the same structural model as in (**a**) (PDB: 6PYH), except that the structure is rotated counterclockwise by 90°. Significant structural destabilizations are shown in red, stabilizations in blue, lack of significant changes in grey, and missing sequence coverage in black. Residues are colored based on differences in deuteration at a single time point (± 2 SD, *p* = 0.02). In both (**a**) and (**b**), IGF-1 molecules are shown as magenta spheres. Of note, residues of the rhesus eIGF1R used for HDX-MS data collection were mapped by BLAST onto the mouse eIGF1R before rendering the HDX-MS results onto the 3D structure of mouse eIGF1R (PDB: 6PYH).

We next assessed the global structural consequences of IGF-1 and VHH-IR5 binding to the soluble eIGF1R using differential bottom-up HDX-MS profiles^33^. This method is extremely sensitive to conformational changes in proteins and has been employed for mapping antibody-epitope interactions^34,35^. The HDX-MS experiments were performed using the fully-glycosylated form of eIGF1R to preserve native eIGF1R conformations, similar to a strategy utilized for the insulin receptor (IR)^36^. Overall, 47% sequence coverage was achieved across 134 peptides (Table 1 and Table S1). Despite the resulting gaps in overall sequence coverage (Fig. S4), HDX-MS data still afforded key insights into the eIGF1R-binding characteristics of both protein ligands (Fig. 2). The HDX responses to the native ligand, IGF-1 (Fig. 2a) are similar to what has been reported previously, validating our experimental approach. For example, conformational stabilizations represented by a decrease in deuteration upon binding were observed at the contact site between the L domain and IGF-1 (residues I9-L16 and L57-L63 specifically), and at the dimer interface between the two L2 domains (L2/L2′, Y417-L424) and the two FnIII-1 domains (FnIII-1/FnIII-1′, R488-F493) (Fig. 2a, blue regions). There was increased deuteration or conformational destabilization for the L2 residues C323-L331, which may be related to the hinging motion of L1-CR away from the L2 domain observed in the full-length IGF1R^23^. An increase in deuteration within the FnIII-2 domain (I598-L611 and Y769-N789) likely results from the disruption of the L1/FnIII-2′ interaction associated with ligand recruitment. Bimodal exchange behaviour in a sequence segment spanning insertion domain residues Y628-D649, first identified by Houde et al.^37^, was also observed here in the uncomplexed, IGF-1-bound and VHH-IR5-bound states of eIGF1R. Inspection of the unlabelled isotopic profile (Fig. S5b) shows that the observed bimodality is not a result of overlapped profiles from unrelated peptides. However, further comparison of the exchange profiles between the three states was not possible due to inherent variability of the deuteration in this region (Fig. S5a).

**Table 1:** Summary of HDX-MS experimental parameters^53^.

**Data set**	Control	IGF-1	VHH-IR5
**HDX reaction details**	PBS, pD = 7.0, 25 °C		
**HDX time course (min)**	1, 3, 10, 60		
**# of Peptides found across all samples**	133		
**Sequence coverage**	47%		
**Average peptide length/redundancy**	11.7/1.7		
**Replicates (biological or technical)**	3 (technical)		
**Repeatability (average standard deviation, %)**	1.6	1.5	1.4
**Significant differences in HDX**	Two-state student T-Test performed at each time point (> 2 SD, *p* value 0.05)		

*All HDX-MS experiments were carried out with recombinant and SEC-purified eIGF1R (Fig. S3).

**Figure 3:**
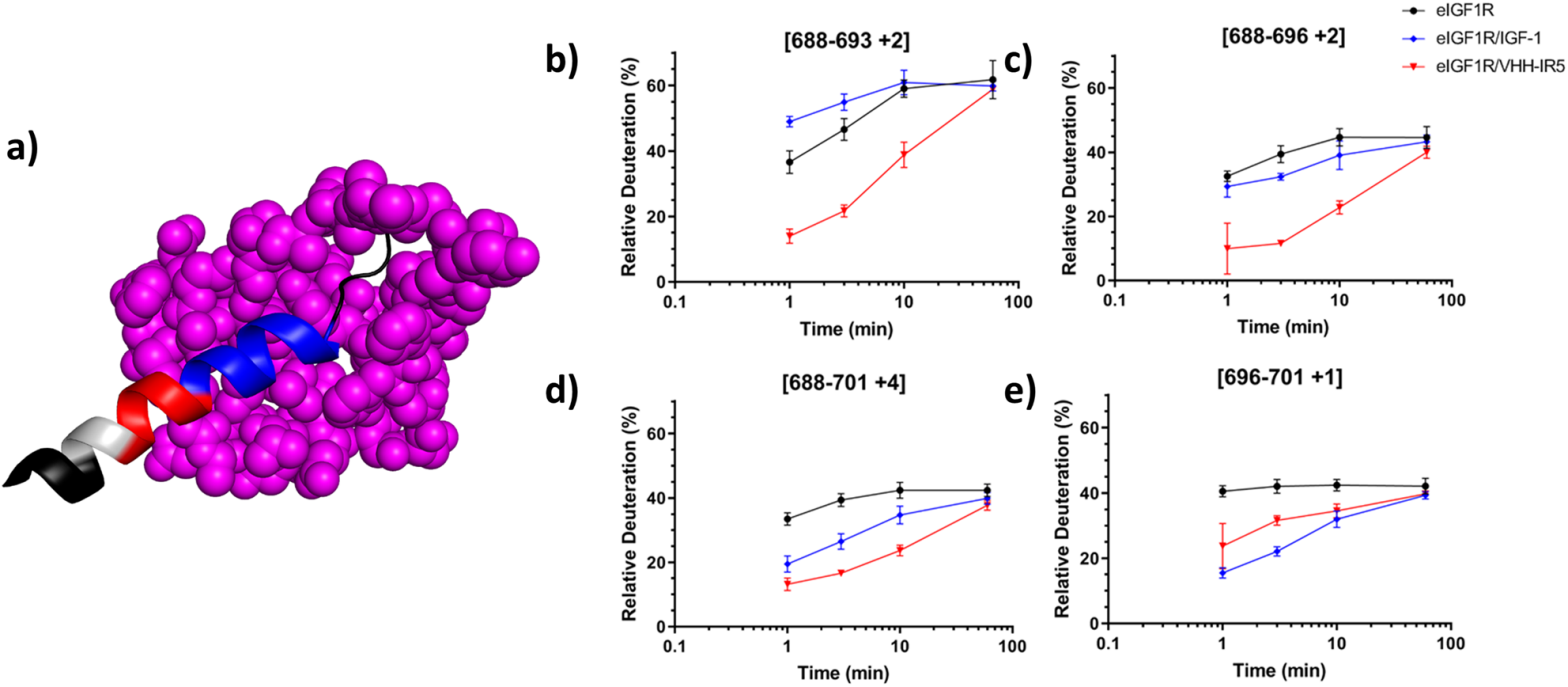
Detailed analysis of the HDX-MS profiles in the IGF1R α-CT region. (**a**) HDX profile of the α-CT’ bound to IGF-1 (magenta) (based on PDB: 6PYH). Residues 684-706 of mouse IGF1R α-CT’ (residues 683-705 in rhesus/human eIGF1R) are shown as a ribbon cartoon. Significant structure destabilization is shown in red and stabilization in blue, while lack of significant changes in grey and missing coverage in black. (**b**–**e**) HDX-MS kinetics of peptides covering the α-CT helix. Data collected in quadruplicate, and error bars represent 1× SD. Deuteration was normalized to theoretical maximum uptake of 45%. Free eIGF1R is shown as black circles, eIGF1R/IGF-1 as blue diamonds, and eIGF1R/VHH-IR5 as red triangles.

Interestingly, many of the dramatic changes in deuteration elicited by IGF-1 were almost entirely absent in response to VHH-IR5 binding (Fig. 2b), suggesting that VHH-IR5 does not induce the same global structural changes in IGF1R. However, both IGF-1 and VHH-IR5 appear to engage the α-CT structural motif located at the C-terminal end of the eIGF1R α-chain, although key differences were observed in the deuteration pattern of the α-CT helix (Fig. 3a) and its immediate surroundings. First, the HDX-MS profile for the IGF-1/eIGF1R interaction (Fig. 3a) closely tracks engagement of the α-CT helix and the associated conformational malleability^38^. Specifically, an increase in deuteration was observed with residues Y688-E693, which suggests that this sequence segment is conformationally more dynamic relative to apo-eIGF1R, despite elongation of the α-CT helix in the IGF-1 bound state^23^. This is followed by progressively stronger reduction in deuteration as peptide coverage shifts towards the C-terminus of the α-CT motif, implying the strongest structural stabilization for residues N694-F701 (Fig. 3b–e). In contrast, binding of VHH-IR5 induced stabilization across residues in the N-terminal region of the α-CT helix (ID residues K690-F701), with the strongest effect localized at residues K690-F692 (Fig. 3b–e). Additionally, VHH-IR5 triggered structural destabilization within the L1 domain (A61-L87) of eIGF1R (Fig. 2b), which is in close contact with the α-CT-helix in IGF1R^23, 24^.

### Section III: epitope mapping of VHH-IR5 using IGF1R insertion domain (ID) fragments containing the α-CT helix

**Figure 4:**
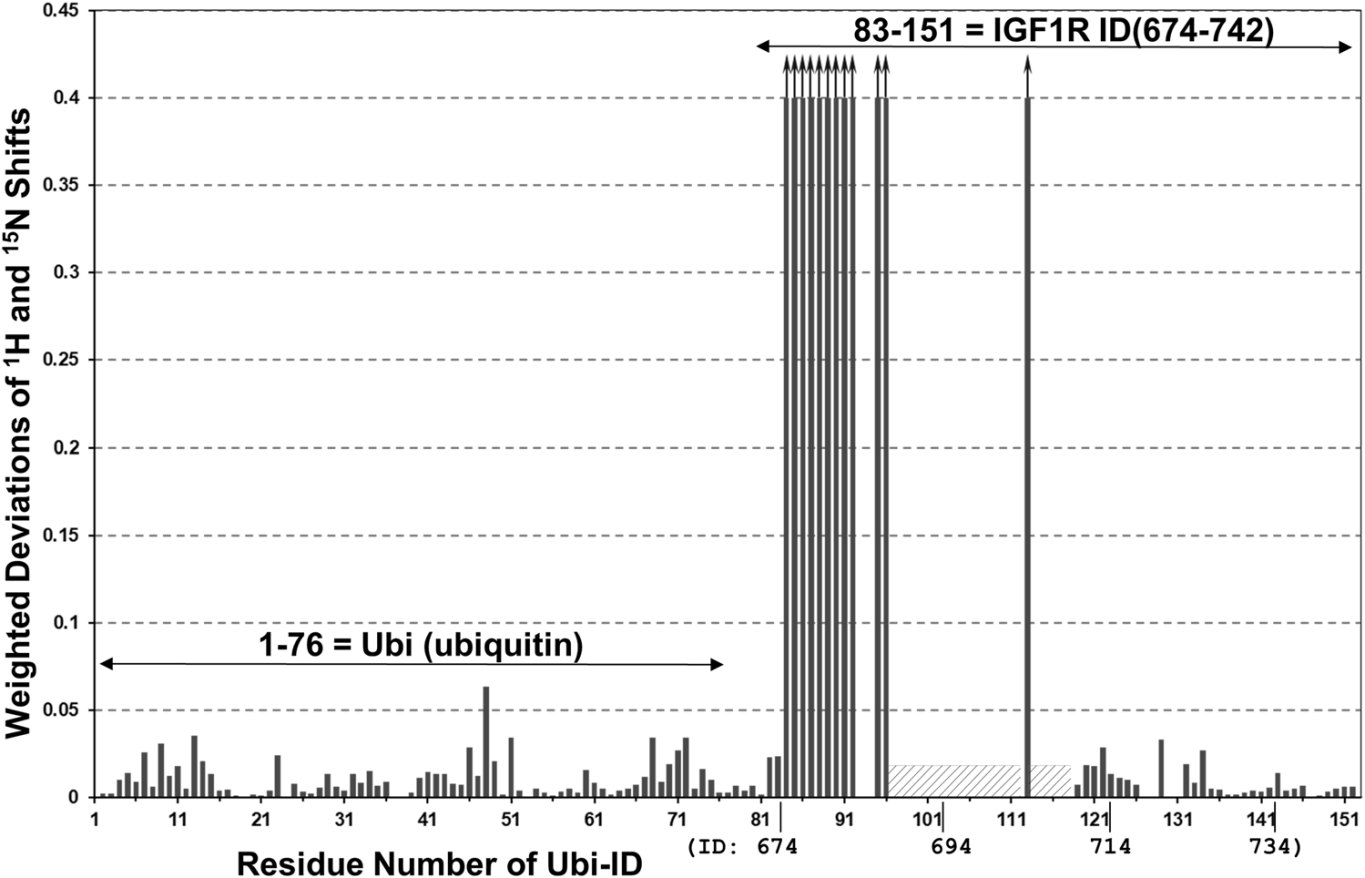
NMR signal perturbations of the ^15^N-labelled Ubi-ID protein by VHH-IR5. Weighted deviations are calculated as the square root of the weighted frequency shifts along both the ^1^H and ^15^N dimensions of the HSQC spectra of the free Ubi-ID as compared to a 1:1 complex of Ubi-ID with unlabelled VHH-IR5 (Fig. S3a) and plotted according to residue-specific assignments achieved for Ubi-ID (Fig. S4). The ubiquitin moiety from 1 to 76 exhibits relatively small differences between the free protein and its complex with VHH-IR5. Some residues of the IGF1R ID 694-742 moiety show pronounced perturbations, especially for T675-E687 (or residues 84-96 of Ubi-ID). Bars with an arrow indicate those residues whose HSQC signals disappeared in the complex of ^15^N-labelled Ubi-ID with VHH-IR5. Hatched boxes indicate that no HSQC signals were found for this region of Ubi-ID (R689-R709), except for R704. HSQC signals of the ID segment re-emerge from residue 119 to 151 (R710-E742), with essentially no responses to VHH-IR5 binding.

In order to further define the role of the α-CT helix motif in ligand engagement, we employed NMR to examine the binding behaviour of VHH-IR5 with IGF1R fragments derived from the insertion domain (Table 2). Two fragments, IGF1R674-742 and IGF1R689-742, in the form, respectively, of Ubi-ID and Ubi-s-ID fusion proteins (Table 2) retain the covalent junction between the extreme C-terminus of the α-chain and the N-terminus of the β-chain in IGF1R that is present in pro-IGF1R^1^. Interestingly, both Ubi-ID and Ubi-s-ID, the latter lacking the N-terminal part of the long α-CT helix, have specific binding with VHH-IR5, as shown by localized perturbations in the ^1^H-^15^N SOFAST-HMQC map of ^15^N-labelled Ubi-ID and Ubi-s-ID by unlabelled VHH-IR5 (Fig. S6). Ubiquitin moieties present at the N-terminus of Ubi-ID and Ubi-s-ID served as an internal control for the interpretation of NMR signal perturbations. The ubiquitin protein is not expected to bind VHH-IR5 and indeed the ubiquitin moiety in the fusion protein showed minimal HMQC signal perturbations (Figs. 4 and S6).

**Table 2:**
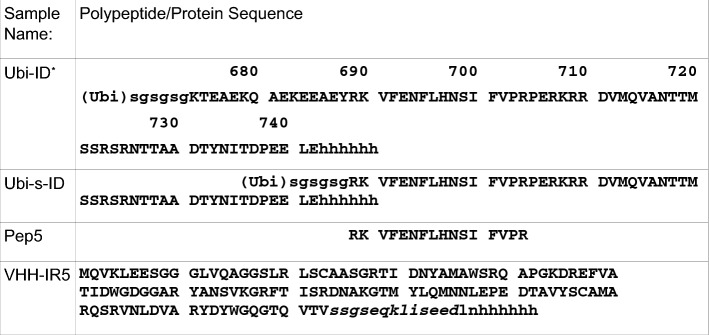
Polypeptide and protein constructs used in NMR and related binding studies.

*(Ubi) denotes the ubiquitin moiety, which has an amino-acid sequence of MQIFVKTLTG-KTITLEVEPS-DTIENVKAKI-QDKEGIPPDQ-QRLIFAGKQL- EDGRTLSDYN-IQKESTLHLV-LRLRGG. Ubi-ID is, therefore, a 157-residue fusion protein and Ubi-s-ID, a 142-residue fusion protein. For Ubi-ID, Ubi-s-ID and Pep5, upper-case letters designate the amino-acid sequence of the uncleaved single-chain human IGF1R precursor^1^. In lower-case letters are the linker sequence, sgsgsg (for Ubi-ID and Ubi-s-ID), and the c-Myc tag ssgseqkliseed (in VHH-IR5) and a His6 tag added for protein expression and purification. All expressed proteins contain an artificial Met residue at the N-terminus added by the *E. Coli* host during protein expression.

The observed NMR HMQC perturbations for the longer ID fragment were localized in the N-terminal region, i.e., residues T675-K683, A686 and E687 (Fig. 4); in other words, further upstream to the α-CT helix characterized by HDX-MS (Fig. 3). Surprisingly, the HSQC map (as compared to SOFAST-HMQC, *vida infra*) of Ubi-ID appears to lack assignable signals for the entire C-terminal region of the α-CT helix, i.e., residues R689-V702 and E706-E709, including residues E706-R709 within an extended segment after the α-CT helix (Fig. S7). These signals are missing at both near-neutral pH 6.8 and pH 5.5 (Fig. S7), despite that this latter lower pH condition normally favors optimal NMR signal intensities. The more sensitive proton-^15^N SOFAST-HMQC experiment^39^ revealed a few additional crosspeaks of very low intensities (Fig. S8a,b), suggesting that the largely invisible region of Ubi-ID (Figs. 4 and S7) must be a consequence of severe line broadening of the NH and/or ^15^N NMR signals of the IGF1R689-709 segment. As well, the SOFAST-HMQC spectrum of Ubi-ID became sharper at an elevated temperature (308 vs. 298 K, Fig. S8c), especially in the spectral region where new SOFAST-HMQC signals (i.e. the observable α-CT residues) are located (Fig. S6). These same SOFAST-HMQC signals also experience more significant changes in NH/^15^N chemical shifts upon temperature elevation (Fig. S8c). Taken together, these NMR lineshape behaviors strongly implicate the α-CT residues as being involved in conformational dynamics such as frame shifts of the α-CT helix (Fig. 3) or other yet-to-be-clarified structural transitions.

**Figure 5:**
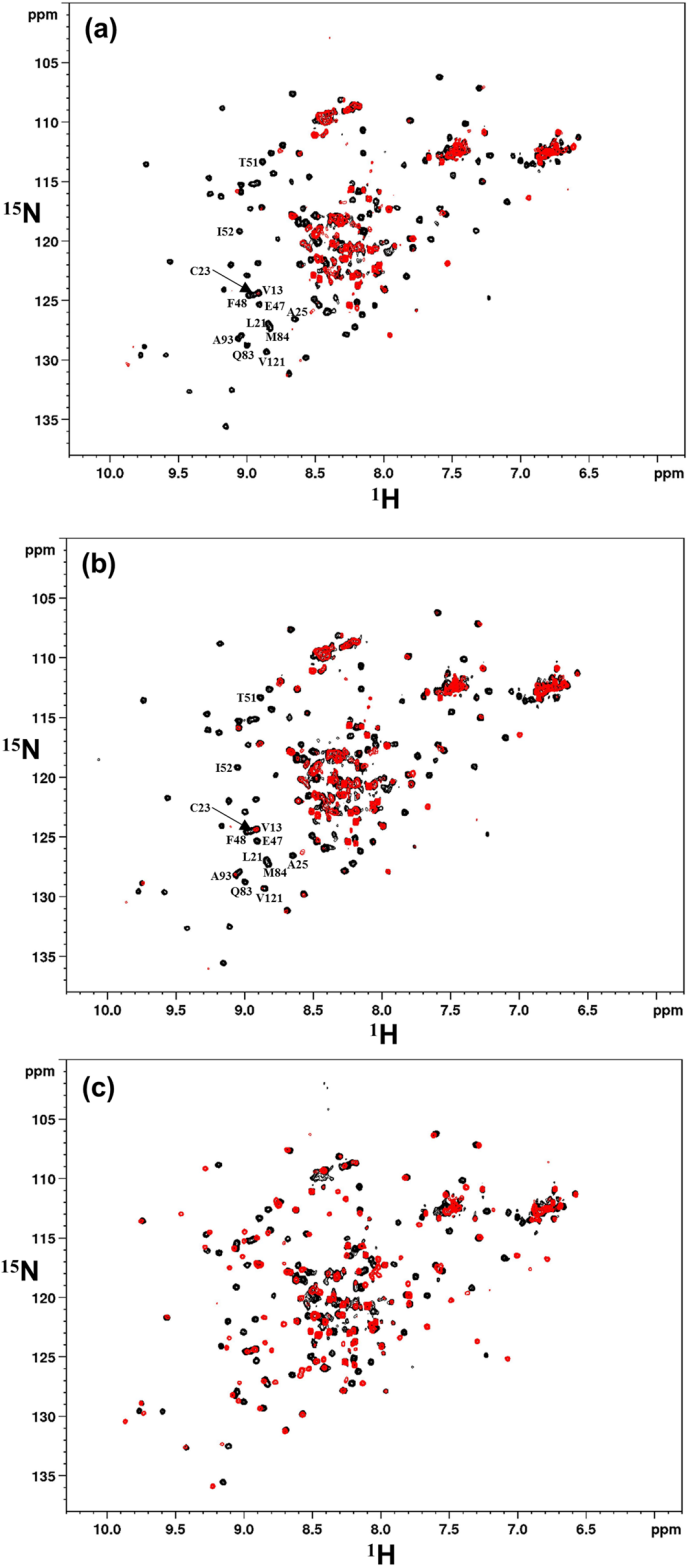
Comparative responses of VHH-IR5 to IGF1R ID fragments. HSQC spectra of ^15^N-labelled VHH-IR5 identify effects of interactions with three unlabelled IGF1R ID fragments. In black are the HSQC spectra of free VHH-IR5 collected immediately before additions of Ubi-ID, Ubi-s-ID and Pep5 while superimposed in red are the HSQC spectra of the sdAb complexes at ~ 1:1 molar ratio. (**a**) spectral comparisons showing the effects of Ubi-ID binding. (**b**) spectra showing the effects of Ubi-s-ID binding. Note that Ubi-ID (**a**) and Ubi-s-ID (**b**) binding induce almost the same perturbations, i.e. disappearances of many HSQC signals of ^15^N-labelled VHH-IR5, especially those of residues T51 and I52 at the beginning of the CDR2 loop and many other residues in the framework region. All perturbed residues were labelled using the resonance assignments of ^15^N-labelled VHH-IR5 (Fig. S5). (**c**) Widespread spectral displacements of ^15^N-labelled VHH-IR5 were produced by binding of the peptide fragment Pep5.

Intriguingly, as many as 10 new SOFAST-HMQC peaks appeared from within the IGF1R689-742 fragment of the shorter Ubi-s-ID construct, which can be traced to the sequence segment without significant HMQC signals in Ubi-ID (Fig. S6). All these re-appeared SOFAST-HMQC signals exhibited specific perturbations in the presence of VHH-IR5. The binding interactions were then examined in complementary experiments using ^15^N-labelled VHH-IR5 titrated with unlabelled Ubi-ID and Ubi-s-ID. Figure 5a and b show that ^15^N-labelled VHH-IR5 responds specifically to binding of Ubi-ID and Ubi-s-ID, in a similar fashion as the ^15^N-labelled Ubi-ID and Ubi-s-ID responding to unlabelled VHH-IR5 (Fig. S6). Despite lacking the N-terminal residues K674-Y688 of the long α-CT helix, binding of the Ubi-s-ID construct perturbs essentially the same HSQC signals of VHH-IR5 as binding to Ubi-ID (Fig. 5a,b). Very importantly, the same set of perturbed residues in VHH-IR5 include some of CDR2, e.g. T51 and I52 and many in the framework region, e.g. V13, L21, A25, E47, F48, Q83, M84, A93 and V121 (Fig. 5a,b), based on the NMR assignments of free VHH-IR5 (Fig. S9).

To further explore the interactions of VHH-IR5 with the α-CT helix, a short peptide derived from the ID region of IGF1R similar to the fragment VFENFLHNSIFVPRPE (α-CT^691-706^) was produced (Pep5, Table 2). Previously, this portion of the α-CT helix was utilized for reconstituting a hybrid IR-IGF1R micro-receptor^40^. More importantly, Pep5 contains all of the amino acids in the α-CT region of IGF1R that were seen to have modified HDX-MS patterns upon VHH-IR5 and/or IGF-1 binding. The synthetic Pep5 (unlabelled) was used here to titrate into ^15^N-labelled VHH-IR5 for selective observations of responses in ^15^N-labelled VHH-IR5. Instead of broadening and the resulting disappearance of many HSQC signals of free VHH-IR5, Pep5 binding to VHH-IR5 led to the appearance of new HSQC signals (i.e., those from the Pep5-sdAb complex) accompanying the disappearance of the HSQC signals of the free VHH-IR5 (Fig. 5c). Such widespread differences between free and ligand-bound proteins are characteristic of slow off-rates and indicative of high-affinity binding interactions in contrast to weaker molecular interactions giving rise to NMR signal broadening (Fig. 5a,b) and disappearance^41^. A smaller peptide fragment of the IGF1R ID such as Pep5 (Table 2), or the related α-CT^691-706^ peptide^40^, very likely has a high binding affinity to VHH-IR5, suggesting that it may represent the linear epitope of this sdAb.

**Figure 6:**
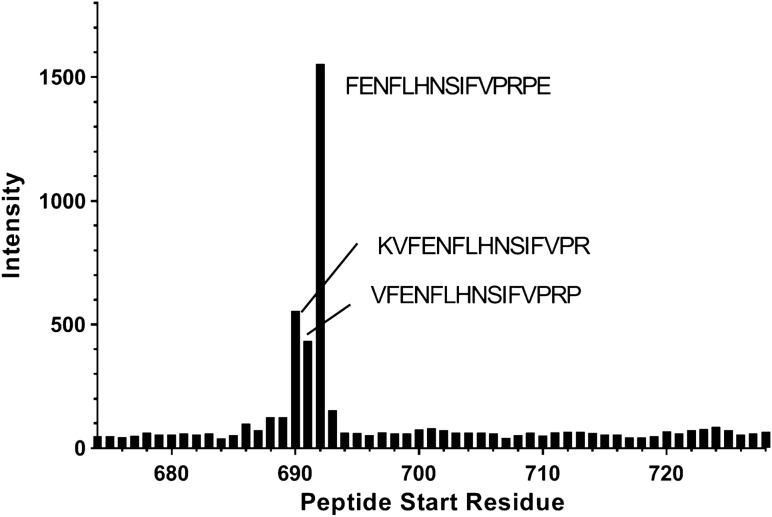
VHH-IR5-binding profile of 15-residue peptide fragments covering IGF1R 674-742. The horizontal axis corresponds to the first residue in each peptide and the vertical axis is the ELISA readout (Table S2) in the PepScan assay^52^. A high level of binding of peptide 692-FENFLHNSIVPRPE-706 (containing R704) should be noted in light of the large NMR signal perturbations of the same Arg residue.

To confirm our results with Pep5, the epitope of VHH-IR5 was further examined using a library of 15-residue peptides that covers the same 674-742 region of IGF1R contained in Ubi-ID (Table 2). Interestingly, the first peptide KTEAEKQAEKEEAEY showed no binding at all to VHH-IR5 (Fig. 6 and Table S2) despite containing all the residues of Ubi-ID with large HSQC signal perturbations (Fig. 4). Binding interactions only become observable for peptides KVFENFLHNSIFVPR, VFENFLHNSIFVPRP, and FENFLHNSIFVPRPE covering IGF1R residues K690-E706, which mirror the strong NMR signal perturbation for residue R704 (Fig. 4). There was essentially no difference between Ubi-ID and Ubi-s-ID for their binding to ^15^N-labelled VHH-IR5 (Fig. 5) since both carry the same sequence K690-E706 of IGF1R (Table 2). NMR signal perturbations in the N-terminal region of the α-CT helix therefore reflects conformational changes induced by VHH-IR5 binding to the epitope segment as represented by Pep5, which, for yet unknown mechanisms, has severely broadened NMR signals as part of the longer ID fragments in Ubi-ID (Fig. S8). This latter experimental NMR observations may be related to HDX-MS results (Fig. 3), outlining significant structural stabilizations of this important region of IGF1R in response to VHH-IR5 binding.

## Discussion

In the search for sdAbs targeting IGF1R, VHH-IR5 has demonstrated the characteristics required for drug delivery across the BBB^21^: efficient BBB transmigration, no detectable impact on the functional activation of IGF1R and no interference with IGF1R/IGF-1 interaction (Fig. 1). The uniqueness of this BBB-crossing sdAb is further highlighted by the nature of the binding epitope of VHH-IR5, revealed here using an array of orthogonal and complementary methodologies. In summary, HDX-MS and NMR results converged on an epitope sequence consisting of residues FENFLHNSIFVPR located near the extreme C-terminus of the IGF1R α-chain (the α-CT). This region of the IGF1R ectodomain demonstrated significant decreases in deuteration in HDX-MS profiles (Fig. 3), equating a structural/conformational stabilization upon binding either driven by allostery or as a direct consequence of binding contacts or both. Of particular interest is the finding that IGF1R fragments containing FENFLHNSIFVPR all retain binding to VHH-IR5 as shown by use of NMR spectroscopy (Figs. 4 and 5). The nature of the VHH-IR5 epitope is further highlighted by two shorter fragments of IGF1R having increased binding affinities for VHH-IR5: RKVFENFLHNSIFVPR (Pep5, Table 2 and Fig. 5) and FENFLHNSIFVPRPE (Fig. 6). These peptide segments terminate, 6 and 4 residues, respectively, before the α-chain C-terminus of IGF1R (Table 2), which is exposed by enzymatic cleavage during IGF1R maturation (Fig. S6)^1^. Taken together, the sdAb VHH-IR5 has a prototypical linear epitope^42^, which is available on the surface of the IGF1R structure, allowing binding of VHH-IR5 to native IGF1R on the cell surface.

At first glance, the identified VHH-IR5 epitope directly overlaps with part of the IGF-1 binding site, residues F692-N694, L696-N698, and I700-F701 identified previously^37,38,43^ and seen here again using HDX-MS (Fig. 3). VHH-IR5 binding therefore necessitates availability of this sequence motif in both apo-IGF1R and in the IGF-1/IGF1R complex since these two ligands do not appear to interfere with each other for IGF1R binding (Fig. 1). As a disulfide-linked homodimer, IGF1R contains two potential binding sites and IGF-1 has been shown to engage only one of these sites on full-length IGF1R at biologically relevant concentrations, as a consequence of negative cooperativity^23,25,26^. Upon binding to IGF1R, IGF-1, along with the L1 domain of one monomer, engages the α-CT' helix of the other monomer, and induces a dramatic structural rearrangement along with the enlongation of the helical structure of the free α-CT toward the N-terminal region (residues 670-681)^23^. For non-competitive binding to be possible, the second α-CT site must remain competent for binding VHH-IR5 and vice versa for IGF-1. Alternatively, both binding sites can be equally available for independent ligation of IGF-1 and VHH-IR5 as shown by SPR evidence of simultaneous IGF-1 and VHH-IR5 interaction with eIGF1R (Fig. 1a). Interestingly, despite dramatic structural rearrangement, the unligated α-CT of the 1:1 IGF-1/IGF1R complex indeed maintains a surface-exposed configuration^23^, leaving this second α-CT available for VHH-IR5 complexation as we observed here for the IGF1R ectodomain (Fig. 1).

The precise mechanism for how VHH-IR5 interacts with the rest of IGF1R without activating downstream signalling events remains largely unknown. However, there are clearly identifiable differences between structural changes of the IGF1R ectodomain in response to IGF-1 and VHH-IR5 binding. The HDX-MS responses in FnIII-1, -2, and L2 of eIGF1R to IGF-1 binding closely mirror the recently-determined symmetric conformation of the IGF1R ectodomain in complex with two IGF-1 molecules^37,38^. Such responses are notably absent upon binding to VHH-IR5. There is, instead, a decrease in deuteration at the L1 domain of the eIGF1R-sdAb complex (Fig. 2b), which suggests structural relaxation of this L1 region subsequent to VHH-IR5 complexation with the α-CT. This same L1 region interacts with the α-CT helix in an apo-form of eIGF1R^38^. In the case of VHH-IR5, its main binding effect is in stabilizing the entire helix and limiting its conformational sampling. Taken together, while the IGF-1 binding elicits more global conformational rearrangements in eIGF1R, VHH-IR5 appears to exhibit a localized binding response through a predominantly linear epitope at the α-CT. VHH-IR5 binding may also be negatively cooperative in the sense that it can only bind a single α-CT site on IGF1R. Since VHH-IR5 alone does not activate IGF1R, VHH-IR5 must induce conformational changes in IGF1R that are different from the large structural shifts induced by IGF-1, such that associations of the IGF1R transmembrane domains and the subsequent receptor auto-phosphorylation cannot take place. Such a distinct mode of receptor engagement by VHH-IR5 may underline the structural basis of the transcytosis capacity of this unique sdAb without a dramatic impact on the normal functions of IGF1R. Future structural investigations are needed, particularly with more complete HDX-MS sequence coverages, for a detailed understanding of the novel sdAb-IGF1R interaction.

### Limitations

This work is focused on assigning the epitope of an IGF1R-specific sdAb VHH-IR5 and comparing the molecular interactions with those observed for the natural ligand IGF-1. Consequently, the ectodomain of IGF1R was selected for the binding studies and for the comparison. There are therefore limitations in the data that prevent detailed assessments of the global architecture of the sdAb-IGF1R complex. Indeed, our HDX-MS data accurately reflect structural shifts in the *bound* α-CT motif reported by Li et al. for a full-length IGF1R^23^. Our HDX-MS findings suggests a limited degree of asymmetry upon IGF-1 binding to the IGF1R ectodomain, which is not in conflict with asymmetric and simultaneous binding of VHH-IR5 and IGF1 in the full length receptor. It is also plausible that the low deuteration content may mask the underlying asymmetry in the HDX profiles^44^. We are unable to explore this question in greater depth within the scope of this current study.

## Methods

### Surface plasmon resonance (SPR)

The binding of IGF-1 and VHH-IR5 to immobilized IGF1R ectodomain (eIGF1R) was determined using BIACORE 3000 (GE Healthcare). Approximately 3000 Resonance Units (RU) of recombinant human eIGF1R (R&D Systems, Cat# 391-GR-050) were immobilized on a sensor chip CM5 after the quality of the eIGF1R was confirmed by size-exclusion chromatography (SEC, see Fig. S1a). Immobilization was carried out at 10 µg/mL in 10 mM acetate at pH 4.0 using the amine coupling kit (GE Healthcare). The remaining reactive sites were blocked with 1 M ethanolamine at pH 8.5. An ethanolamine blocked surface was used as a reference. Binding studies were carried out at 25 °C in 10 mM HEPES, pH 7.4 containing 150 mM NaCl, 3 mM EDTA and 0.005% surfactant P20 (Polyoxyethylenesorbitan, GE Healthcare). Various concentrations of SEC-purified IGF-1 and VHH-IR5 (Fig. S1b,c) were flowed over the immobilized eIGF1R and reference surfaces at 20 uL/min. The concentrations employed for VHH-IR5 were 0.75, 1, 2.5, 5, 5, 7.5 and 10 nM, while for IGF-1 binding, they were 10, 25, 50, 100 and 250 nM. The SPR co-injections (saturation of the surface with injection of analyte 1, followed by injection of a mixture of analyte 1 + analyte 2) utilized VHH-IR5 at 10 nM and IGF-1 at 250 nM. Surfaces were regenerated, after binding of VHH-IR5 with 10 mM glycine at pH 2.0 with a contact time of 24 s. No regeneration was used for IGF-1 binding experiments. Data were analysed with BIAevaluation 4.1 (GE Healthcare). The data was fit to a 1:1 binding model without further attempts at quantifying the binding stoichiometry (Text S1).

### Cell-based IGF1R functional assay

The receptor functional assay was carried out using the PathHunter eXpress IGF1R kit (DiscoverX), which uses engineered HEK293 cells expressing a (Pro-Link or PK-) tagged IGF-1 Receptor and an Enzyme Acceptor (EA-) tagged SH2 domain. Upon receptor activation, EA-SH2 binds to the phosphorylated PK-IGF1R and reconstitutes an active β-galactosidase enzyme which hydrolyzes a substrate to generate a chemiluminescent readout.

Cells were thawed and plated in a 384-well white wall clear bottom plate (Greiner, North Carolina, USA) at 20,000 cells/well in the cell plating 17 reagent media. After a 24-h incubation at 37 °C in an atmosphere with 5% CO2, cells were treated with IGF-1 (R&D, Minneapolis, USA) (with concentrations varying from 0.03—2000 nM), VHH-IR5 (0.03–6000 nM) or 3 nM IGF-1 + VHH-IR5 (0.03–6000 uM) for 180 min at RT. The chemiluminescent substrate was added and cells were further incubated at RT for 60 min. The resulting luminescence was measured using the CLARIOstar plate reader (BMG LABTECH, Ortenberg, Germany).

### Expression and purification of the IGF1R ectodomain

Codon-optimized cDNAs (Genscript) for the rhesus IGF1R ectodomain (eIGF1R, residues 1-902) with a C-terminal His-tag was subcloned into pTTTM109 expression vector. A pool of CHO cells (CHOBRI55E1-JN, proprietory to The National Research Council of Canada) was initiated and the CHOBRI TM55E1 pool was selected with methionine sulfoximine for approximately two weeks. A fed batch production was then performed in optiFlasks. The eIGF1R protein was purified by immobilized metal ion affinity chromatography followed by size-exclusion chromatography in 1× PBS (HiPrep 26/60Superdex 200) (Fig. S3a). Eluting fractions were monitored by SDS-PAGE (Fig. S3b), pooled, concentrated to 10.17 mg/mL and stored at − 80 °C.

### Peptides and expression of isotope-labelled proteins for NMR Spectroscopy

Fusion proteins of IGF1R ID fragments, Ubi-ID and Ubi-s-ID, were constructed by adding the sequence of human ubiquitin (Ubi) to the N-terminus of the two IGF1R fragments (Table 2). The DNA sequences were codon-optimized for *E. coli*, synthesized and inserted into the pD441-SR expression vector (Atum). The plasmids were electroporated into a modified BL21 *E. coli* host (F–*ompT lon hsdSB,rB*^*-*^*,mB*^*-*^)*gal dcm* [*malB*+]K-12(λS) *rhaB elaD*) and selected with 50 µg/mL kanamycin. The expression plasmid for VHH-IR5 was constructed similarly using the normal BL21 *E. Coli* and contained a c-Myc tag and a His_6_ tag linked to the C-terminus of the sdAb using a SSGS spacer (Table 2). Further details for VHH-IR5 production are provided in Text S2 (Supplementary Materials). 

Protein expression was induced in the presence of kanamycin by addition of 1 mM IPTG at 24 °C overnight. Uniform ^15^N- and ^13^C/^15^N-labeled proteins were obtained by growing the cells in defined media containing 2 g/L d-glucose (U-13C6-99%, Cambridge Isotope Laboratories, Inc) and 1 g/L ammonium sulfate (15N2-99%, Cambridge Isotope Laboratories, Inc) as the sole carbon and nitrogen source, respectively. Cell pellets were re-suspended in ~ 150 mL lysis buffer (50 mM sodium phosphate, 300 mM NaCl, pH 8), sonicated on ice for 2 min, and clarified at 10,000 rpm at 4 °C for 30 min. The clarified lysate was mixed with 3 mL Ni–NTA resin by gently vortexing, washed with 100 mL lysis buffer + 20 mM imidazole, and eluted with lysis buffer + 250 mM imidazole. Eluted protein was dialyzed into 50 mM sodium phosphate, 50 mM NaCl at either pH 6.8 or pH 5.5. The peptide Pep5 (Table 2) was synthesized chemically using the solid-phase method (CanPeptide Inc., Pointe-Claire, Quebec, Canada) and purified by use of HPLC. Identities of the purified peptide Pep5 and Ubi-fusion proteins were confirmed by mass spectrometry: Pep5—theoretical FW of 2003.3 versus experimental mass of 2003.2; Ubi-ID—theoretical FW of 17,937.03 versus experimental intact mass of 17,938; Ubi-s-ID—theoretical FW of 16,172.17 versus experimental intact mass of 16,173. The integrity of the bacterially-expressed VHH-IR5 (Text S2) was verified by ^1^H-^15^N NMR HSQC and by sequence-specific residue assignments using 3D NMR spectra of ^15^N/^13^C-labelled VHH-IR5 protein.

### Bottom up HDX-MS

HDX-MS was performed by first mixing 20 μM eIGF1R with 25 μM ligand (IGF-1 or VHH-IR5) in PBS at a 1:1 volume ratio. Labelling was initiated by adding 3 μL of 90% D_2_O (0.1× PBS, pD 6.9) to 3 μL of an IGF1R-complex at 19 °C. The labelling reaction was quenched by the addition of a 54 μL ice-cold solution of 250 mM TCEP, 2 M Guanidine, 100 mM glycine–HCl at pH 2.0. Quenched and deuterium-labelled samples were immediately injected into a 20 μL sample loop (at 20 pmol eIGF1R/25 pmol ligand). Samples were non-specifically digested online with a Poroszyme Immobilized Pepsin Cartridge (2.1 × 30 mm, Thermo Scientific) at room temperature for 1.5 min at 75 μL/min, and desalted at 400 μL/min with a C18 PepMap100 trap at 1 °C (1 × 5 mm, 5 µm, Thermo Scientific) in mobile phase A (0.23% Formic Acid in water, pH 2.55). Peptides were eluted from a BioBasic-18 (50 × 0.32 mm, 5 µm, Thermo Scientific) column with a separation gradient spanning 10–35% mobile phase B (0.23% Formic Acid in Acetonitrile) over 20 min. Samples were injected into Q-TOF Premier mass spectrometer (Waters) in quadruplicate technical replicates, and deuteration assignment and statistical analysis of individual timepoints (2 × SD, *p* = 0.02) were achieved using MS Studio^45^. Unlabelled eIGF1R was injected and analyzed by data-dependent acquisition, and peptides were identified with Mascot. Further experimental details can be found in Table 1. Sequence alignment of the rhesus eIGF1R used for HDX-MS on to the mouse eIGF1R was performed with BLAST^46^ prior to displaying the HDX-MS data on the 3D structure (PDB: 6PYH) of mouse eIGF1R (Figs. 2 and 3). The sequences of rhesus and human eIGF1R are highly homologous and completely in frame with each other.

### NMR spectroscopy

All NMR experiments were carried out at a sample temperature of 298 K on Bruker Avance-III 600 MHz and 800 MHz NMR spectrometers equipped with a 5 mm PATXI sample probe and Z-field gradient accessories. Assignments of the ^1^H-^15^ N correlations (i.e. HSQC or HMQC) spectra for Ubi-ID (Table 2) were achieved using 3D HNCA, HN(CO)CA, CBCA(CO)NH, HNCACB, HN(CA)CO, and HNCO spectra^47^ collected at 600 MHz. The NMR sample was prepared from a 0.5 mM uniformly ^15^N-/^13^C-labeled Ubi-ID dissolved in a buffer that was 50 mM in sodium phosphate and 50 mM in NaCl at pH 5.5 followed by the addition of 10% D2O. The SOFAST-HMQC^39^ pulse sequence with 0.3 s recycle delay was used to record 2D ^1^H-^15^N correlation spectra of Ubi-ID and Ubi-s-ID (Table 2) titrated with varying concentrations of the unlabelled sdAb VHH-IR5.

NMR experiments for VHH-IR5 (Table 2) resonance assignments were carried out at 800 MHz with a 0.3 mM uniformly ^15^N-/^13^C-labeled VHH-IR5 dissolved in the NMR buffer (see above) at pH 6.8 followed by the addition of 10% D_2_O. Multi-dimensional heteronuclear NMR data, i.e. HNCA, HN(CO)CA, HNCACB, CBCA(CO)NH, HN(CA)CO, and HNCO were collected using pulse sequences from the Bruker pulse sequence library. HN(CA)CO and HNCO experiments were carried out with a non-uniform (sparse) sampling (NUS) schedule generated using the jittered rejection sampling algorithm^48^. These NUS schedules were individually optimized for the ^13^C and ^15^N dimensions, separately, each using a 50% of sparsity with denser sampling at short evolution times, resulting in, for example, a total of 32 sampled data points from a uniform (Nyquist) grid of 64. Water suppression was achieved using gradient selection for all 3D NMR experiments. The 3D HN(CA)CO and HNCO spectra were processed from time-domain data matrices after filling the NUS data along with extrapolation in each of the indirect dimensions using an in-house implementation of the Convex-Accelerated Maximum Entropy Algorithm (CAMERA)^49^. The filled 3D NUS-NMR data matrices were processed using the same processing parameters as for complete data matrices, but leaving out linear prediction, by use of NMRPipe^50^. All other 3D NMR spectra were processed using NMR Pipe with linear prediction for the two evolution dimensions. Analysis of 3D NMR spectra and resonance assignments were achieved using nmrView^51^.

### Fragment scanning of the IGF1R Insertion Domain

A total of 55 overlapping peptides of 15 residues in length were derived from the 674-742 sequence segment of human IGF1R using the PepScan procedure^42,52^. Binding activities of the surface-immobilized peptides were quantified through ELISA using an anti-His antibody specific to a His-tag present in VHH-IR5 (Table 2). Concentration of VHH-IR5 was 10 μg/mL in all loading solutions.

## Supplementary Information

Supplementary Information.

Supplementary Table S1.

Supplementary Table S2.

## Data Availability

All data generated or analyzed during this study are included in this published article.

## Abbreviations

HDX: Hydrogen–deuterium exchange
HDX-MS: Hydrogen–deuterium exchange mass spectrometry
NMR: Nuclear magnetic resonance
HSQC: Heteronuclear single quantum coherence
HMQC: Heteronuclear multiple quantum coherence
SOFAST-HMQC: Band-selective optimized flip-angle short transient HMQC
HNCA, HN(CO)CA, CBCA(CO)NH, HNCACB, HN(CA)CO, and HNCO: A set of heteronuclear 3D NMR experiments for backbone resonance assignments of ^15^N- and ^13^C-labelled proteins
NUS: Non-uniform (sparse) sampling
SPR: Surface plasmon resonance
RU: Resonance unit
PDB: Protein data bank
SD: Standard deviation
CDR: Complementarity-determining region
IGF1R: Type-1 insulin-like growth factor receptor
eIGF1R: The ectodomain of IGF1R
IGF-1,2: Type-1/2 insulin-like growth factor (or insulin-like growth factor 1, 2)
BBB: Blood brain barrier
sdAb: Single-domain antibody
α-CT: The C-terminus of the α-subunit of IGF1R
L1 and L2: Leucine-rich domains 1 or 2
L1-CR: L1 constant region
FnIII-1,2,3: Fibronectin type III domains 1,2,3 of IGF1R
RTK: Receptor tyrosine kinase
IR: Insulin receptor
IRR: Insulin receptor–related receptor
CNS: Central nervous system
RMT: Receptor mediated transcytosis
TfR: Transferrin
LDL: Low-density lipoprotein
SH2: Src homology 2 domain
EA: Enzyme acceptor
ID: Insertion domain
HEK293: Human embryonic kidney cell line 293
CHO: Chinese hamster ovary cells
RT: Room temperature
PBS: Phosphate Buffered Saline
SDS-PAGE: Sodium dodecyl sulfate–polyacrylamide gel electrophoresis
IPTG: Isopropyl β-D-1-thiogalactopyranoside
TCEP: Tris(2-carboxyethyl)phosphine
EDTA: Ethylenediaminetetraacetic acid
HPLC: High-performance liquid chromatography
