# Supplementary material for: Defining the epitope of a blood–brain barrier crossing single domain antibody specific for the type 1 insulin-like growth factor receptor
Source: Sci Rep. 2021 Feb 19;11:4284. doi: 10.1038/s41598-021-83198-w (PMC7896052; doi:10.1038/s41598-021-83198-w)
Supplement: Supplementary file 1 — Supplementary Information. [file 41598_2021_83198_MOESM1_ESM.pdf]

Supplementary Materials for Joey Sheff et al & Feng Ni:

Defining the Epitope of a Blood-Brain Barrier Crossing Single Domain Antibody Specific for the Type 1 Insulin-Like Growth Factor Receptor

Joey Sheff[1], Ping Wang[2], Ping Xu[2], Melanie Arbour[2], Luke Masson[2], Henk van Faassen[1], Greg Hussack[1], Kristin Kemmerich[1], Eric Brunette[3], Danica Stanimirovic[3], Jennifer J. Hill[1], John Kelly[1], & Feng Ni[2,*]

Human Health Therapeutics Research Centre, National Research Council Canada, [1]100 Sussex Drive, Ottawa, Ontario, Canada K1A 0R6, [2]6100 Royalmount Avenue, Montreal, Quebec, Canada H4P 2R2 and [3]1200 Montreal Road, Ottawa, Ontario, K1A 0R6

Content: Two sections (Text S1 and S2) of supplementary texts along with 9 supplementary Figures S1-S9, 3 supplementary references and two supplementary Tables S1 and S2 (in separate files).

Text S1: Comments on deduction of binding stoichiometry from SPR data.

Interpreting stoichiometry from SPR data in the current assay format is a considerable challenge. The immobilized IGF1R ectodomain (IGF1R) is a homodimer that may present 1 or 2 binding sites for the ligand (IGF-1 or the single-domain antibody VHH-IR5) depending on positive or negative cooperativity between the two. To accurately deduce binding stoichiometry, one we would need to have a 100% active IGF1R surface. Hypothetically, if 100 RUs of IGF1R were immobilized, the theoretical maximum binding response (R_{max}theo) for binding of IGF1 to one site might be 100 RUs and to two sites 200 RUs. That assumes that IGF1R is 100% active and that binding of one ligand (e.g. IGF-1) does not block nor prevent access of a second. In practice, the observed maximum binding response (R_{max}obs) is routinely much lower in SPR assays, typically in the range of 20 – 75% of the theoretical maximum, due to multiple factors including inaccessibility of epitopes, inactive or denatured protein populations, and other impacts from the coupling chemistry. Therefore, in the hypothetical example, if a maximum binding response of R_{max}obs = 100 RU was observed, it could be interpreted as IGF-1 binding at one site on IGF1R (100% active surface), or it could be interpreted as IGF-1 binding at two sites on IGF1R (50% active surface).

The R_{max}obs from IGF-1 binding IGF1R (65 RU; Figure S2) did not exceed the R_{max}theo of even one binding site, suggesting that there may be only one site on IGF1R occupied by IGF-1 but for the reasons stated above one cannot reach that conclusion without uncertainty. A more appropriate method to determine binding stoichiometry would be ITC (isothermal calorimetry) experiments, which can be pursued in the future when adequate quantities of IGF1 and IGF1R will become available. For this study, however, the lack of quantitative information on binding stoichiometry does not affect the validity of the competition SPR assays between two different ligands, namely IGF-1 and VHH-IR5, and the conclusions drawn based on the data obtained therein (Figure 1).

Text S2: A detailed procedure for bacterial expression and purification of VHH-IR5 for NMR spectroscopy.

The protein sequence of VHH-IR5 (Table 2) was codon optimized for *E. coli* expression. The gene synthesis and insertion into the pJ401 expression vector were outsourced to Atum (1140 O'Brien Drive, Suite A, Menlo Park, CA 94025 USA, https://www.atum.bio). The plasmid was extracted from the solid support (dried sterile filter paper) using 10 mM Tris buffer (pH 8.5) and transformed immediately into BL21 *E. coli* (F^{-} *ompT lon hsdS$_B$(r$_B^-$, m$_B^-$) gal dcm [malB$^+$]*$_{K\text{-}12}$(λ^S)) by heat shock. The transformants were selected on Animal Product Free (APF) LB agar containing 50 µg/mL of kanamycin. One colony was used to inoculate 5 mL of APF LB + 50 µg/mL of kanamycin, which was incubated overnight at 37°C with 250 rpm agitation. The next day, 1 mL of culture was mixed with 0.5 mL 50% (w/v) glycerol, and stored at -80°C before use.

For VHH-IR5 production, bacterial cell stocks transfected by the VHH-IR5 expression plasmid were inoculated into 1 mL of the LB media containing 50 ug/mL kanamycin and incubated at 37 °C. After 6 hours, the cells were diluted 50-fold into the LB/kana media and incubated at 37 °C overnight, The resulting cells were then diluted 50-fold into the

LB/kana media and incubated at 24 °C until A_{600} = 0.8 ~ 1.0, when protein production was induced by the addition of IPTG to a final concentration of 1 mM in the cell culture incubated overnight at 24 °C. Cell pellets were collected by centrifugation (using a spinning rate of 4K for 20 min at 4 °C) and processed (see below) for protein purification.

Uniform ^{15}N- and $^{15}N/^{13}C$-labeled VHH-IR5 were obtained by growing the cells in a minimal media containing 2g/l of glucose as the sole carbon source and 1g/l of ammonium sulfate as the sole nitrogen source. The minimal media also contained M9 salts, 1 mM $MgSO_4$, 5mg/l thiamine, 100 uM $CaCl_2$, and 50ug/ml kanamycin and sterilized by filteration before use.

Cell pellets were resuspended in 100~150 mL of a lysis buffer (containing 50 mM NaPi 300 mM NaCl at pH 8), sonicated on ice for a total time of 2 min (on = 10s, off = 15s, power = 6) with the lysis debris spun down by centrifugation (at 10K rpm at 4 °C for 30 min). The clear supernatant was mixed with 3 mL of the Ni-NTA resin by gentle shaking. After 30 min, the mixture was loaded into a column and washed by 100 mL of the lysis buffer plus 20 mM imidazole. The protein was eluted using the lysis buffer plus 250 mM imidazole, until the OD_{280} was low. The elution was dialyzed by a buffer of 50 mM NaPi and 50mM NaCl at pH 6.8 or pH 5,5, each time the concentration of imidazole being diluted by 50-fold. After 3 times of buffer exchange, the purified VHH-IR5 was concentrated using a Centricon apparatus (Amicon Inc.) with a molecular weight cuttoff of 8K.

Figure S1: Size-exclusion chromatography (SEC) characterization of IGF-1, the human IGF1R ectodomain and VHH-IR5. Chromatography columns used were: Superdex-200 Increase 10/300 GL for IGF1R and Superdex S75 10/300 GL for IGF1 and the sdAb IGF1R-5 (abbreviated as VHH-IR5 in the main text). SEC runs were carried out at a temperature of 25°C with a flow rate of 0.8 mL/min in the buffer of 10 mM HEPES at pH 7.4 containing 150 mM NaCl, 3 mM EDTA and 0.005% surfactant P20. The quantities of proteins injected were 10 µg for IGF1R, 250 – 300 µg for IGF1 and VHH-IR5 (labelled here as IGF1R-5) in injection volumes of approximately 100-150 µL.

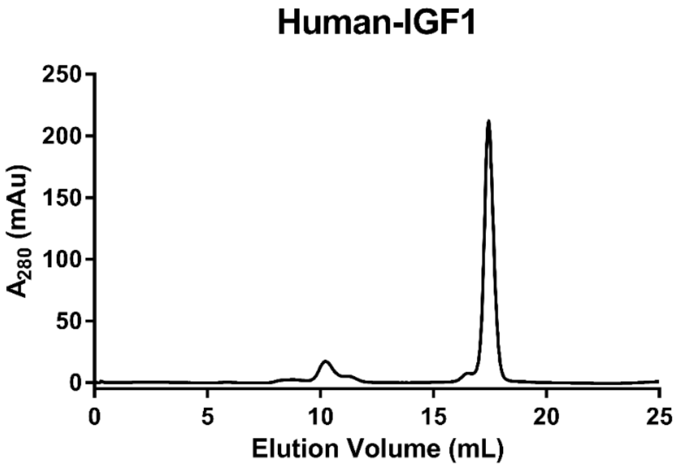

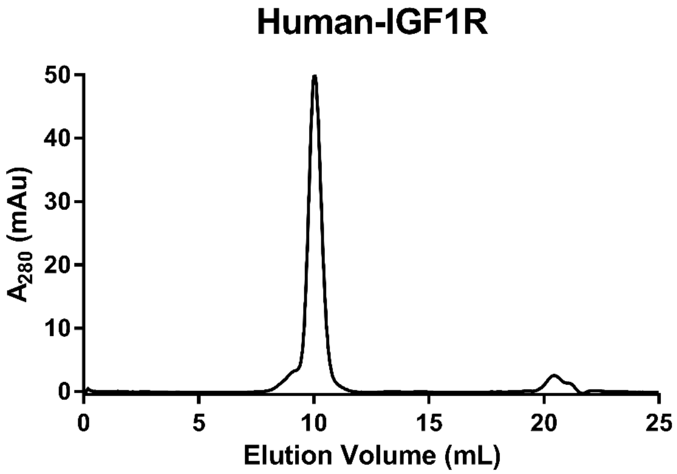

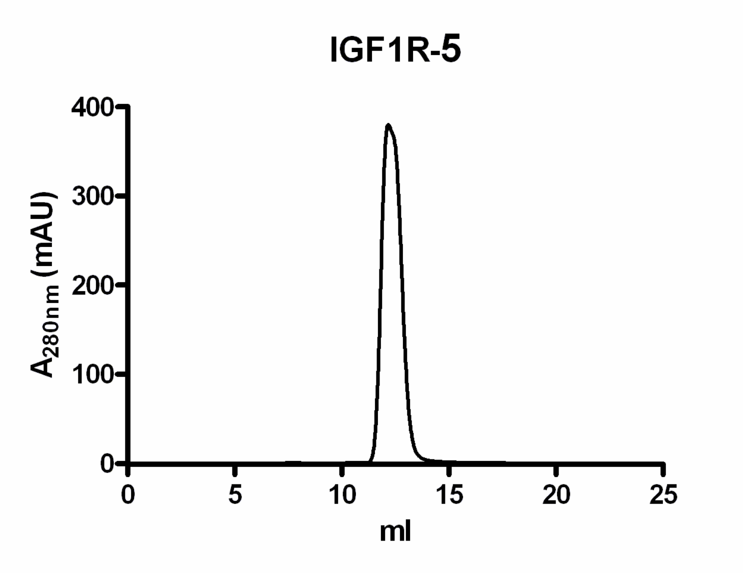

IGF1R-5
A280nm (mAU)
400
300
200
100
0
0
5
10
15
20
25
ml

Figure S2: SPR binding studies of IGF-1 and IGF1R-5 (i.e. VHH-IR5) with surface-immobilized IGF1R and human insulin receptor ectodomains***.**

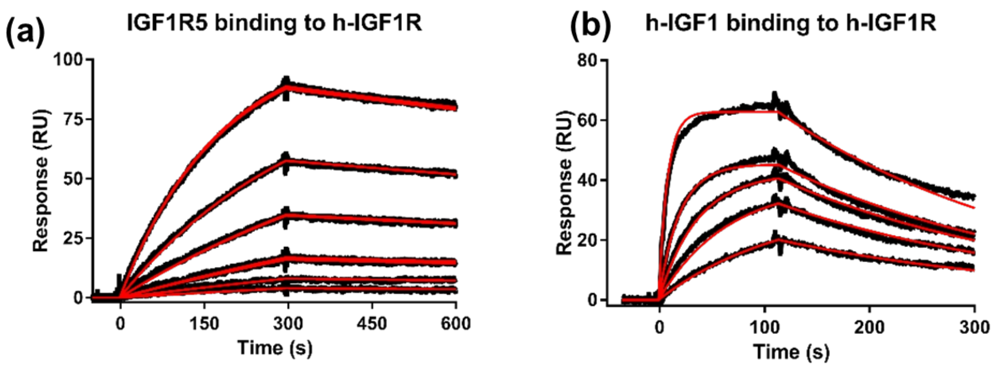

	Rmax (RU)	ka (1/Ms)	kd (1/s)	KD (M)
**VHH-IR5**	115	5.33e5	3.44e-4	6.45e-10
**IGF-1**	65	4.65e5	8.24e-3	8.24e-9

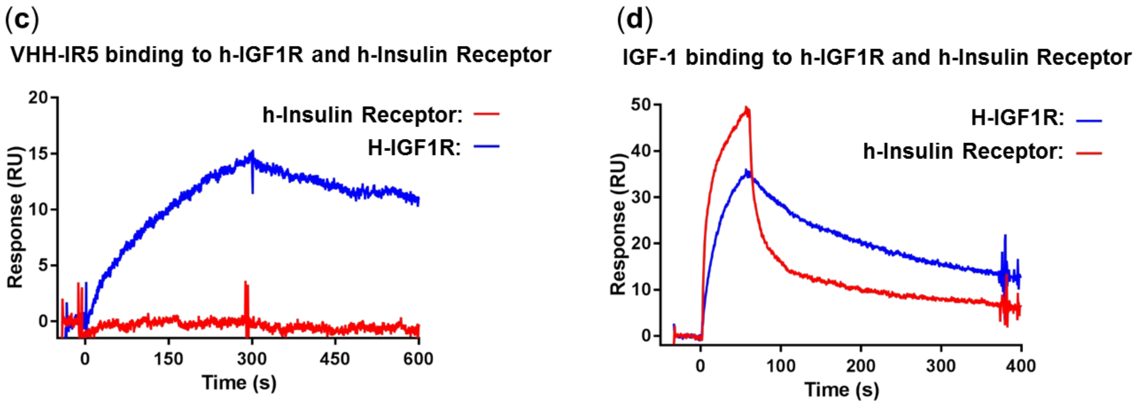

* For Figure S2c and S2d, approximately 4000 RUs of human insulin receptor (R&D Systems, Cat# 1544-IR/CF; His28-Arg750 alpha subunit & Ser751-Lys944 beta subunit, with a C-terminal 10-His tag) were amine coupled on a CM5 sensor chip in 10 mM sodium acetate buffer, pH 4.5. Experiments were performed on a Biacore 3000 instrument (GE Healthcare) in HBS-EP buffer (10 mM HEPES, pH 7.4 containing 150 mM NaCl, 3 mM EDTA and 0.005% surfactant P20) at 25°C. At a flow rate of 20 µL/min, VHH-IR5 (1 nM) was injected over IGF1R and human insulin receptor surfaces for 300 seconds and allowed to dissociate for 300 seconds. HBS-EP buffer was used to regenerate surfaces. IGF-1 (100 nM) was injected over the insulin receptor and IGF1R surfaces for 60 seconds and allowed to dissociate for 600 seconds. HBS-EP buffer was used to regenerate surfaces.

Figure S3: (a) Size-exclusion chromatography profile obtained with the HiPrep 26/60 Superdex 200 column in 1x PBS and **(b)** Coomassie SDS-page of eIGF1R after purification by immobilized metal ion affinity chromatography.

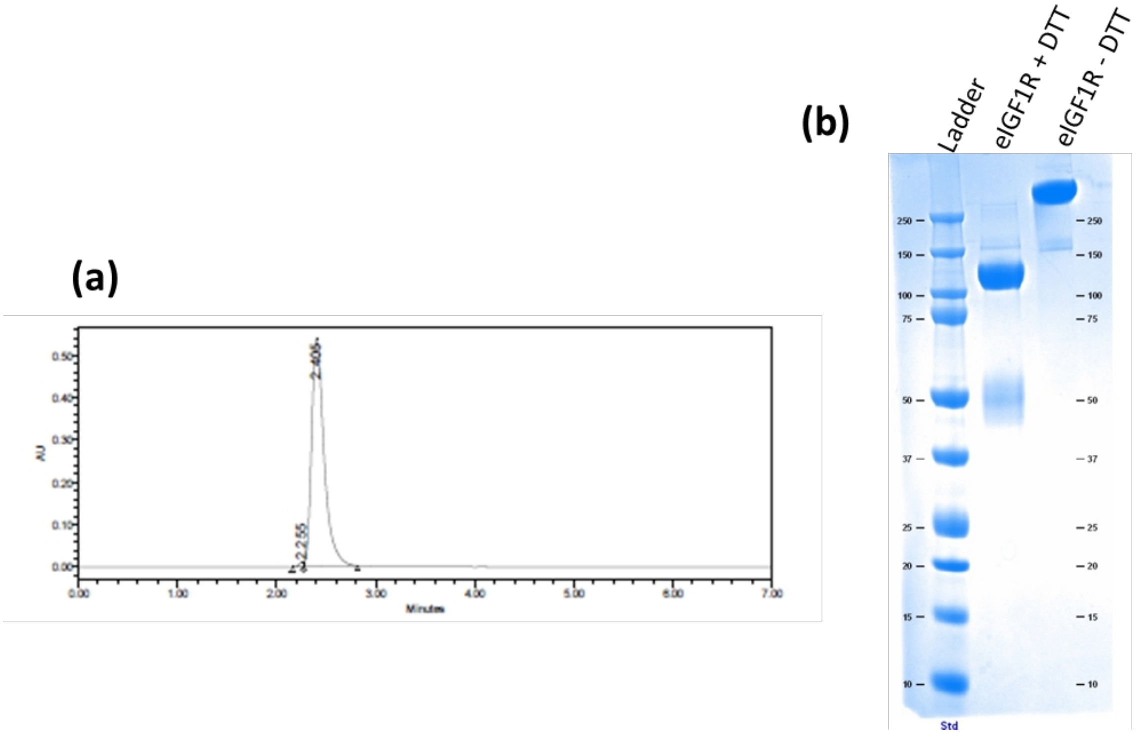

Figure S4. Validated HDX-MS coverage of eIGF1R. Rectangles represent individual peptide features, and predicted N-linked glycans are highlighted by red squares. Map was created with MS tools[1]. Digest conditions and database search parameters are outlined in the Materials and Methods section.

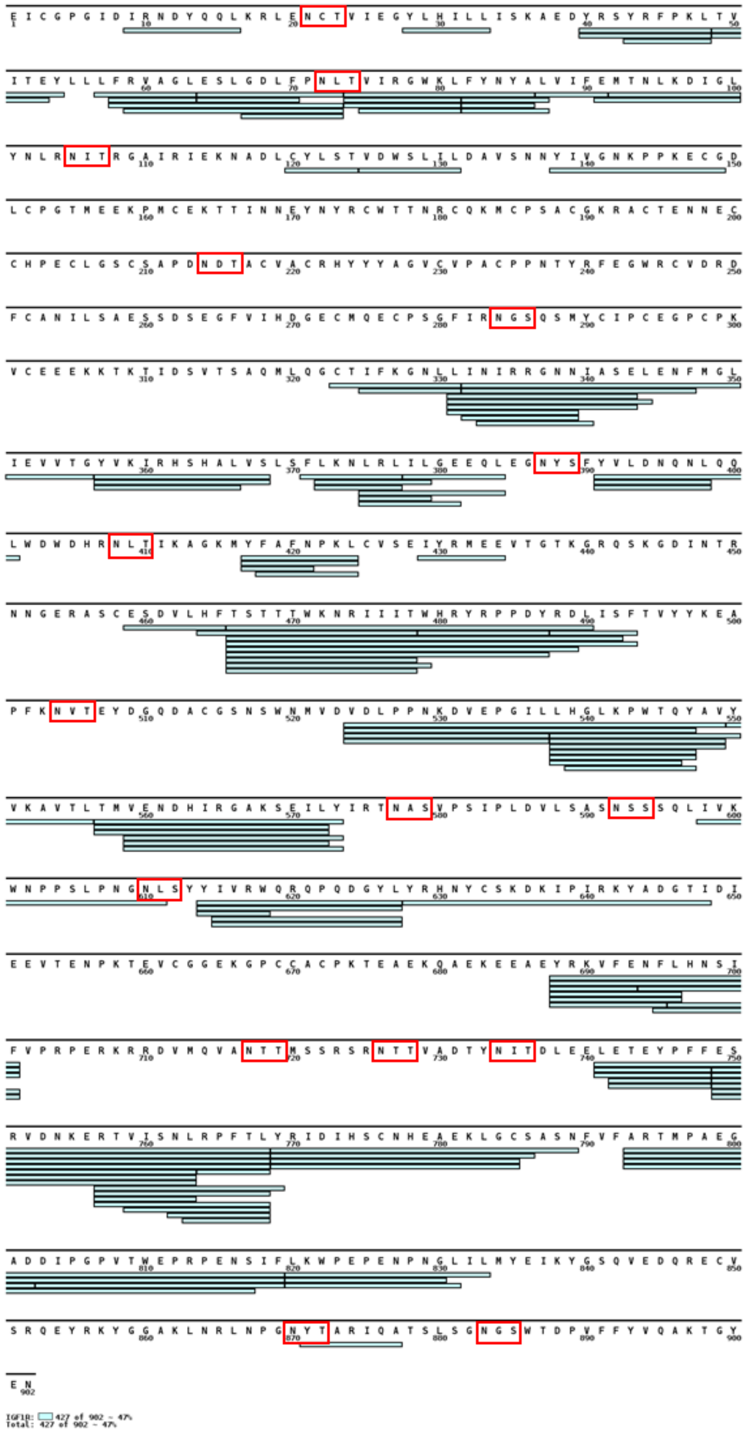

Figure S5. (a) Bimodal isotopic profile expansion observed in peptide 628-YRHNYCSKDKIPIRKYADGTID-648. Screenshots were captured from MS Studio.[2] Isotopic peaks are annotated with experimental m/z, and theoretical undeuterated profile is shown as shaded boxes. **(b)** Undeuterated profile showing no spectral overlap.

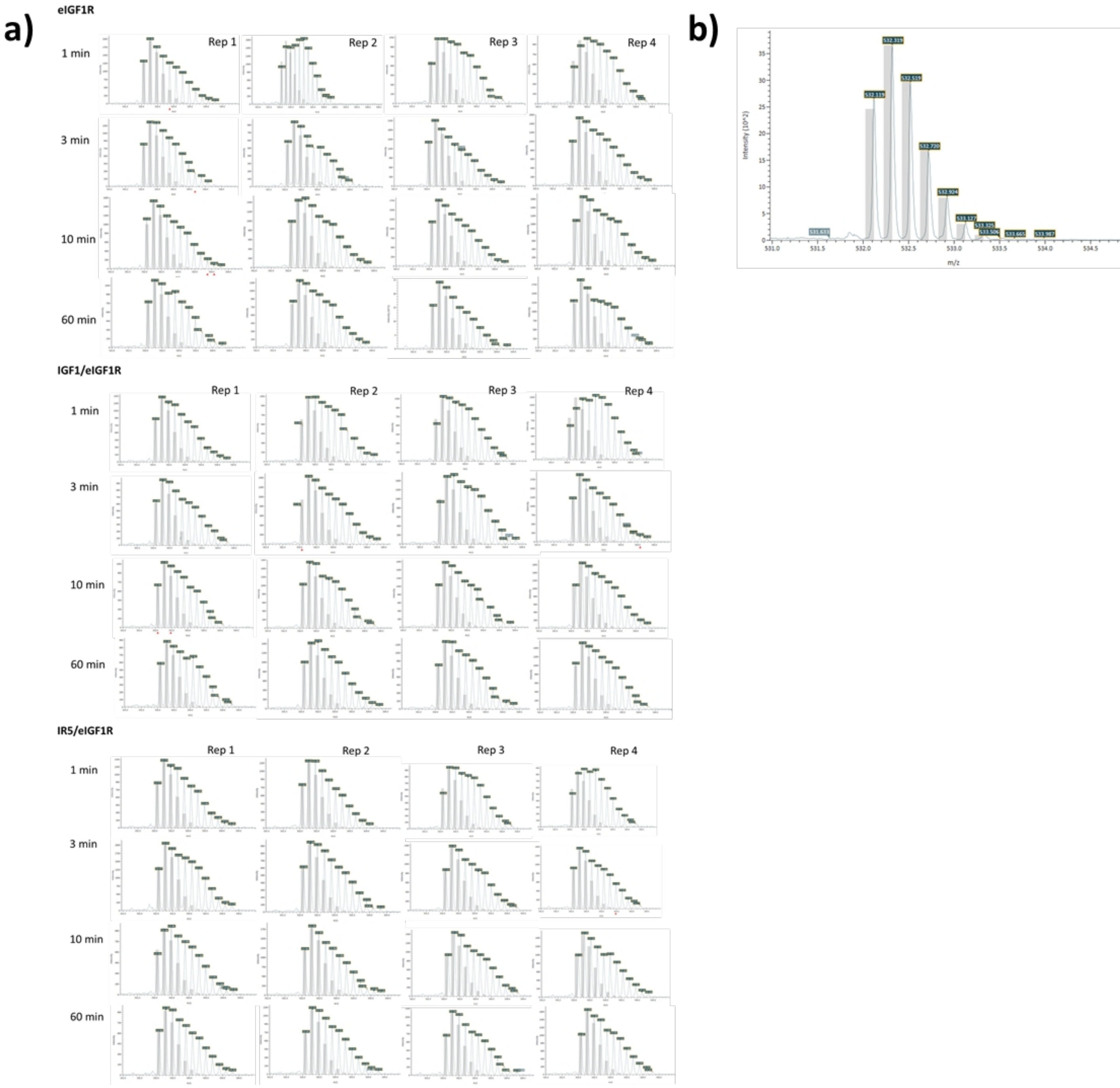

Figure S6. Binding Interactions of IGF1R ID fragments with VHH-IR5 followed by amide H-^{15}N SOFAST-HMQC *. Experimental conditions were 50 mM Na-phosphate/50 mM NaCl at pH 5.5 and 298 K.

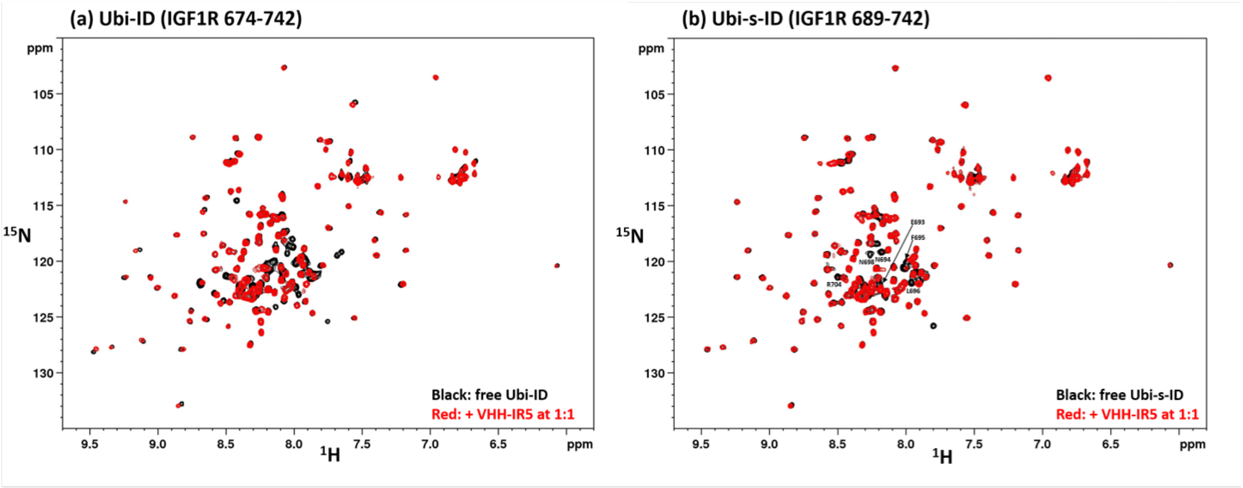


```
        680        690        700        710        720        730        740
ID:   KTEAEKQ AEKEEAEYRK VFENFLHNSI FVPRPERKRR DVMQVANTTM SSRSRNTTAA DTYNITDPEE LE
s-ID:                 RK VFENFLHNSI FVPRPERKRR DVMQVANTTM SSRSRNTTAA DTYNITDPEE LE
```

Furin cleavage site

* Both polypeptides ID and s-ID have the sequence numbering of the IGF1R precursor and both retain an intact peptide bond between residue R710 and D711. This peptide bond is cleaved by furin (or furin-like proteases) during the maturation of IGF1R, which exposes the C-terminus of the α chain (i.e. the α-CT) with a free carboxylate at residue R710. SOFAST-HMQC is used here for titration experiments to maximize the intensity of NMR cross-peaks with line broadening effects. HMQC signals in Ubi-s-ID (Figure S6b) perturbed by VHH-IR5 binding were traced, through spectral comparison, to the R689-E706 region that are largely missing in the assigned HSQC spectrum of Ubi-ID (Figure S7. Colored (red) residues in the sequences of ID and s-ID are those In the R689-E706 segment that can be assigned for ID (Figure S7) and for s-ID (Figure S6a).

Figure S7. Assigned ^{1}H-^{15}N HSQC spectrum of the Ubi-ID fusion protein at pH 5.5 and 298 K *.

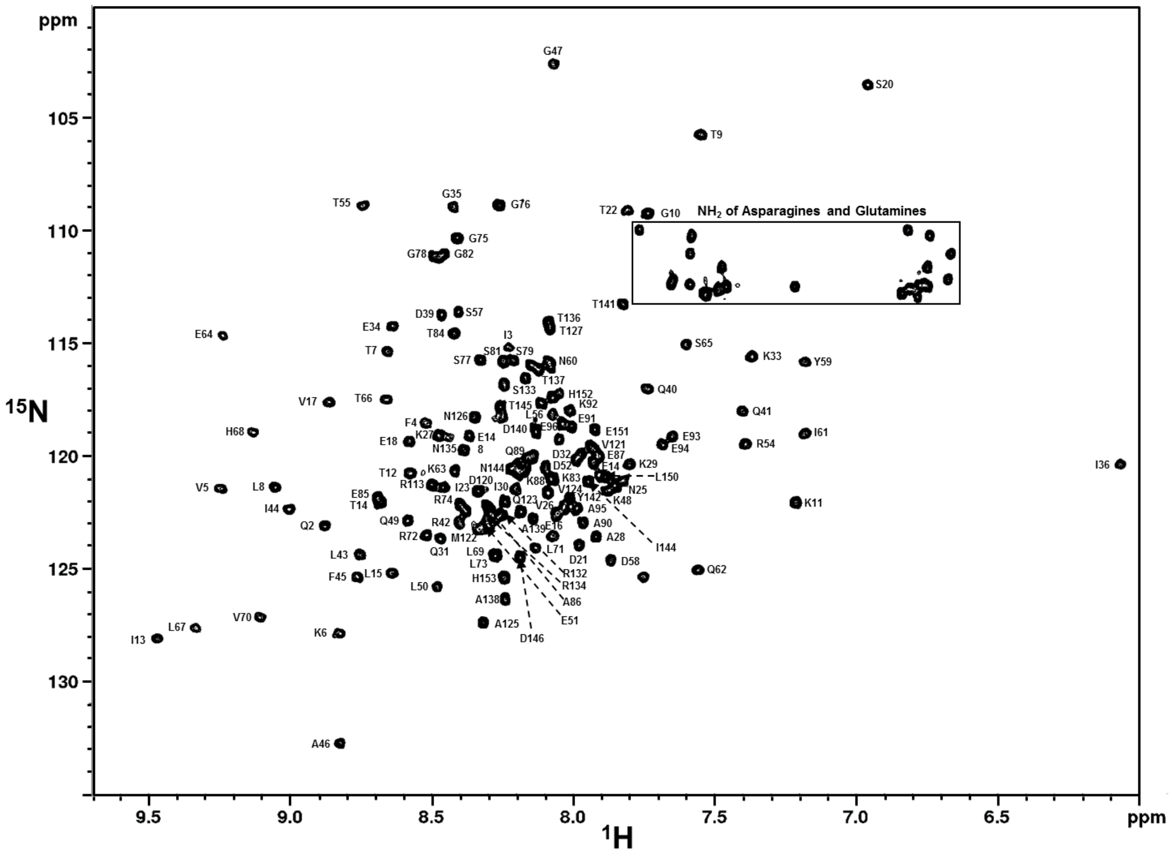

*Corresponding HSQC spectrum at pH 6.8 is essentially the same (not shown) without any additional HSQC crosss peaks at this higher pH condition. The sequence numbering shown here follows that of the Ubi-ID fusion protein, i.e. with IGF1R K674 starting at Ubi-ID residue K83 (see also Figure 4 of main text).

Figure S8. Comparison of the HSQC and SOFAST-HMQC spectrum* of the Ubi-ID (a) Ubi-s-ID (b) fusion proteins at pH 5.5 and 298 K. Panel c is a comparison of the SOFAST-HMQC spectra of Ubi-ID acquired at two different temperatures, at 298 K (in black) and at 308 K (in margenta). The zoomed regions in the insets correspond to the area of the whole spectra indicated by dashed rectangles.

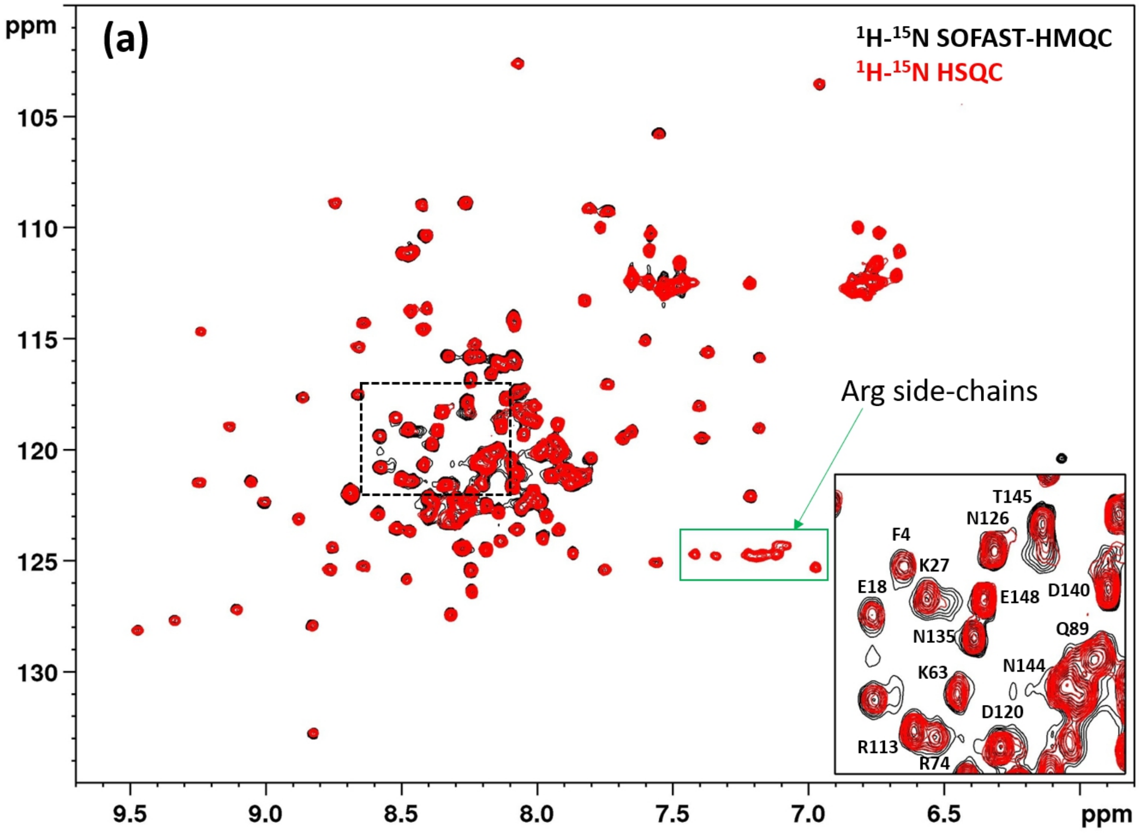

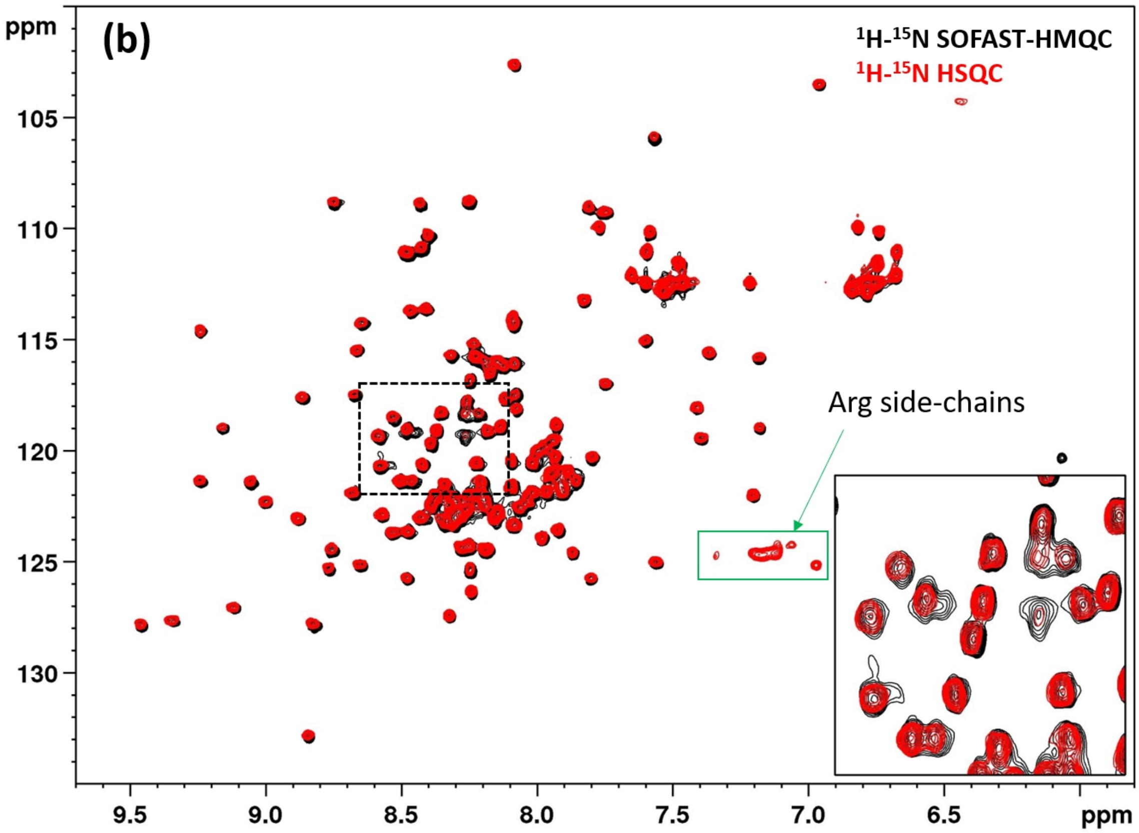
(b)
1H-15N SOFAST-HMQC
1H-15N HSQC
Arg side-chains
ppm
105
110
115
120
125
130
9.5
9.0
8.5
8.0
7.5
7.0
6.5
ppm

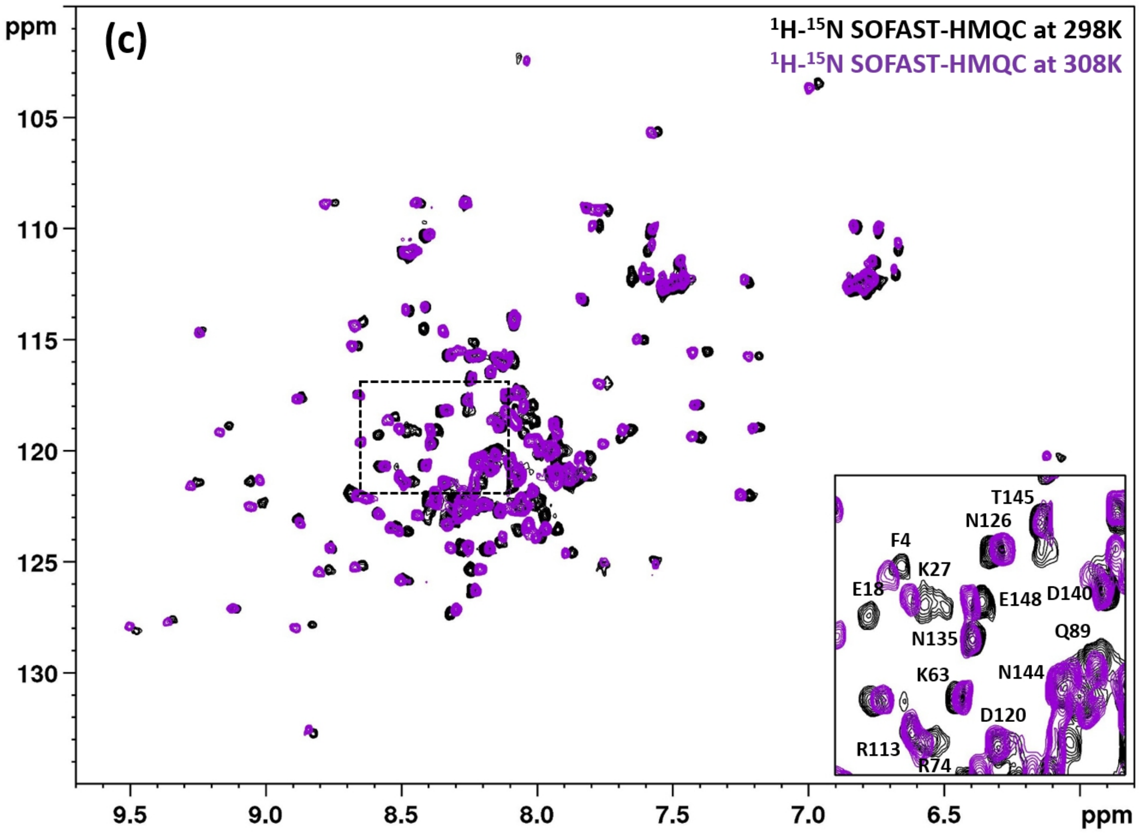

* The sensitivity of SOFAST-HMQC is superior to HSQC[3], which led to the appearance of additional cross peaks with low signal intensities in the SOFAST-HMQC spectra. Shown in red are the same HSQC spectra as that in Figure S7 while the superimposed black spectra were recorded with the SOFAST-HMQC pulse sequence[3], leading to increased signal intensities for broader crosspeaks of residues with shorter amide proton and/or ^{15}N transverse relaxation times. HSQC signals from arginine sidechains are indicated for Figures S8a and S8b since these signals are not observable in SOFAST-HMQC experiments[3].

Figure S9: Assigned ^{1}H-^{15}N HSQC Spectrum of VHH-IR5 at pH 6.8 and 298 K

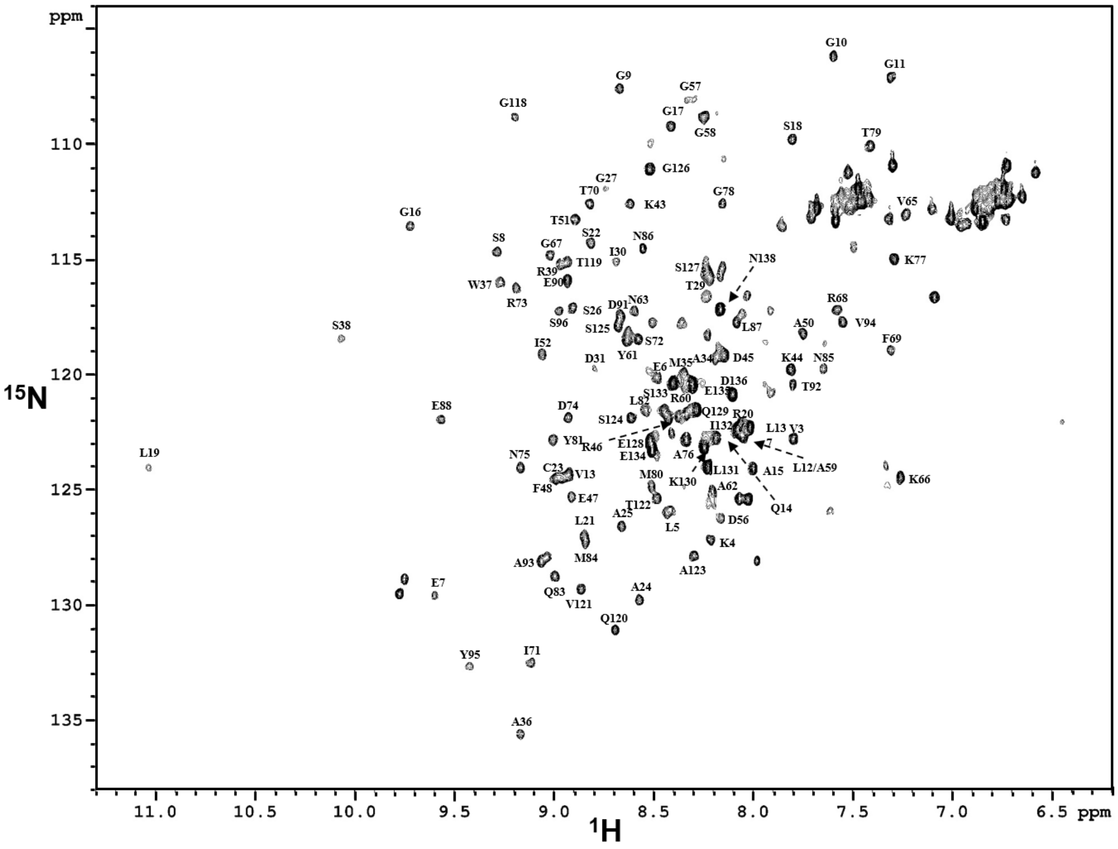

Supporting References

1. Kavan D, Man P. MSTools - Web based application for visualization and presentation of HXMS data. Int J Mass Spectrom [Internet] 2011; 302:53–8. Available from: http://dx.doi.org/10.1016/j.ijms.2010.07.030

2. Rey M, Sarpe V, Burns KM, Buse J, Baker CAH, van Dijk M, Wordeman L, Bonvin AMJJ, Schriemer DC. Mass Spec Studio for Integrative Structural Biology. Structure [Internet] 2014 [cited 2019 Nov 14]; 22:1538–48. Available from: https://www.sciencedirect.com/science/article/pii/S0969212614002585?via%3Dihub

3. Schanda, P., Kupce, E. & Brutscher, B. SOFAST-HMQC experiments for recording two-dimensional heteronuclear correlation spectra of proteins within a few seconds. J Biomol NMR 33, 199-211 (2005).
